# Whole-Brain Evaluation of Cortical Microconnectomes

**DOI:** 10.1523/ENEURO.0094-23.2023

**Published:** 2023-10-25

**Authors:** Kouki Matsuda, Arata Shirakami, Ryota Nakajima, Tatsuya Akutsu, Masanori Shimono

**Affiliations:** 1 Graduate Schools of Medicine, Kyoto University, 53 Kawaramachi, Shogoin, Sakyo-ku, Kyoto 606-8507, Japan; 2 Bioinformatics Center, Institute for Chemical Research, Kyoto University, Gokasho, Uji, Kyoto 611-0011, Japan; 3 Graduate School of Information Science and Technology, Osaka University, 1-5 Yamadaoka, Suita-shi, Osaka 565-0871

**Keywords:** cortex, functional networks, network, nonuniformity

## Abstract

The brain is an organ that functions as a network of many elements connected in a nonuniform manner. In the brain, the neocortex is evolutionarily newest and is thought to be primarily responsible for the high intelligence of mammals. In the mature mammalian brain, all cortical regions are expected to have some degree of homology, but have some variations of local circuits to achieve specific functions performed by individual regions. However, few cellular-level studies have examined how the networks within different cortical regions differ. This study aimed to find rules for systematic changes of connectivity (microconnectomes) across 16 different cortical region groups. We also observed unknown trends in basic parameters *in vitro* such as firing rate and layer thickness across brain regions. Results revealed that the frontal group shows unique characteristics such as dense active neurons, thick cortex, and strong connections with deeper layers. This suggests the frontal side of the cortex is inherently capable of driving, even in isolation and that frontal nodes provide the driving force generating a global pattern of spontaneous synchronous activity, such as the default mode network. This finding provides a new hypothesis explaining why disruption in the frontal region causes a large impact on mental health.

## Significance Statement

This study reports the results of a systematic comparison of the nonuniformity of neural interaction networks, which obtained from electrophysiological measurements of the activity of hundreds of neurons, in 16 regions selected to cover a broad range of the brain cortex. As a result, we observed prominent properties in the deeper layers of the frontal, such as strong connections with other layers, indicating a high capacity to drive other neurons. The fact that this experiment was performed in acute slices indicates that these properties are not driven by any region outside of them. Therefore, this property suggests that the frontal region is a node that drives the macroscopic spatial pattern of spontaneous activity.

## Introduction

The brain is an organ with very nonuniformly connected components working together as a network. Extracting any systematic rule about such nonuniformity in terms of internal characteristics of local components may provide important insights about how the brain processes information in parallel across different scales, such as cellular scale and anatomic scale.

### Focus on cortex

Within the brain, the neocortex is an evolutionary novelty and an important component of the brain that supports the high intelligence of mammals, including humans (Kaas, 2020).

At first glance, in the neocortex, roughly similar circuits are arranged throughout the entire cortical sheet. However, although there is some degree of similarity, there is also a certain degree of uniqueness (DeFelipe et al., 2002; Nelson, 2002; Douglas and Martin, 2004). In fact, in the brain of a mature animal, each cortical region is assigned a distinct set of functions. To perform different functions, individual brain regions must either receive different inputs from the outside via the inter-regional connections or/and have different local circuits within them.

### Inter-regional similarities and differences of cortical microcircuits

In the past, detailed comparisons of the structure of local circuits in different regions have been mainly made among a few cortical areas (Nelson, 2002). It has been suggested that cortical local circuits share a common design and constitute a “canonical circuit” (Douglas et al., 1989; Miller, 2016). At the same time, each region also has a distinct profile of connectivity (Harris and Shepherd, 2015; De Biase and Bonci, 2019; Batiuk et al., 2020). In other words, each area can have systematic differences according to the spatial distances and topological distances of their connections (DeFelipe, 2002; Wang, 2020).

Corroborative evidence has come from comparisons of cell density and cortical thickness in several regions (Fulcher et al., 2019). Studies in the broader mammalian species have also shown that cell density varies across cortical regions, a finding that is being rapidly elucidated not only by classical immunostaining (Collins et al., 2010; Herculano-Houzel et al., 2013; Keller et al., 2018), but also by emerging transparency techniques (Hama et al., 2011; Chung et al., 2013; Susaki et al., 2014). Systematic rules for the relationship between connections between brain regions and cell density are also becoming clearer (Herculano-Houzel et al., 2013; Shimono, 2013).

Despite the widespread interest and substantial volume of research in this field, there still exist few global and quantitative comparisons across different brain regions on how information is internally exchanged among >100 neurons in local neural circuits, based on experimental data. In particular, there are almost no studies that have made a wide comparison between brain regions, stepping into the topological differences in functional connectivity. Therefore, if we can extract the rules of “differences among individual brain regions,” such information is expected to be a source of significant benefits not only for brain modeling but also for, in a wide range of aspects such as contrast with other nonuniformity indicators, understanding of disease states, etc.

Approaches to exploring the topology of connections between hundreds or more neurons have evolved into a research field called the microconnectome (Schröter et al., 2017). Our past studies have also focused on a single brain region to study the microconnectome, and have repeatedly evaluated and improved the technology (Shimono and Beggs, 2014; Nigam et al., 2016; Kajiwara et al., 2021).

This present study extended the technique to all cortical regions of the mouse, and evaluated how each cortical brain region differs especially in terms of the topologies of functional local circuits, and other basic physiological parameters, such as firing rate and layer thickness. Specifically, we conducted an analysis focusing on the prefrontal cortex. This is because the prefrontal cortex forms networks with other brain regions and controls the flow of information, enabling adaptive behaviors. For instance, it regulates various cognitive processes such as information integration, decision-making, planning, and behavioral inhibition (Miller and Cohen, 2001; Bicks et al., 2015; Fuster, 2015). Similar regions to the prefrontal cortex are also found in mice, suggesting that certain higher-order cognitive functions are regulated in mice as well (Uylings et al., 2003). Thus, investigations into the specificity of the prefrontal cortex in mice are being conducted from both cross-species and species-specific perspectives (Morishima and Kawaguchi, 2006; Carlén, 2017). Then, we evaluated density and functional interconnections from active neurons, something that cannot be captured by applying staining technique to dead brain slices.

## Materials and Methods

### Physiologic experiments

**Figure 1.**
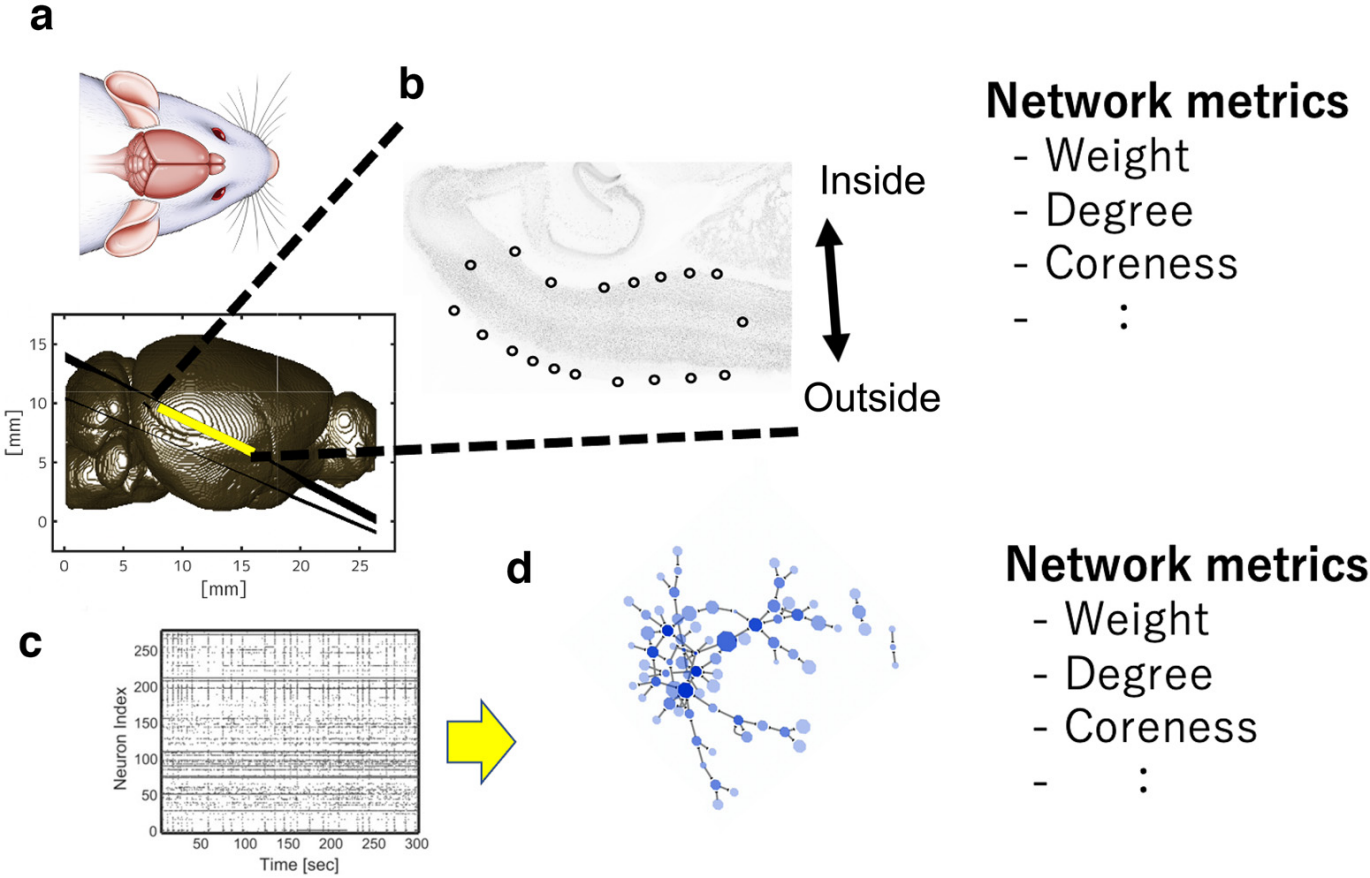
Scheme of the whole analysis. ***a***, An example of a mouse brain magnetic resonance (MR) images. We cut out acute slices at the location of the lines, and measured electrical activity from them, and then immunostained them. ***b***, An example of immunostaining. The cortical area surrounded by black circles indicates the area where electrophysiological measurements were also performed. ***c***, Electrophysiological measurements provide a time series of spikes in the activity of hundreds of neurons. ***d***, From the time series, directional interactions were inferred and effective networks were constructed. We evaluated not only basic physiological metrics but also various metrics of the effective networks. Extended Data Figure 1-1 provides additional information for this figure.

The present study acquired acute brain slices from 16 groups of cortical regions of the cerebral cortex and simultaneously measured electrical activity from a large number of neurons within the brain slices (Fig. 1*a*; Table 1). The experimental protocols we employed, which included 3D scanning, immunostaining, and electrical activity measurements, were consistent with those outlined in previously published studies. These protocols, including detailed explanations, have been demonstrated in a video journal (Ide et al., 2019; Kajiwara et al., 2021; Shirakami et al., 2022). All animal experiments were performed according to animal experiment protocols approved by the Kyoto University Animal Committee. All mice used were female C57BL/6J mice (*n* = 37, three to five weeks old, *n* ≥ 2 per group).

**Table 1:** Definition of 16 regional groups, and number of mice used for individual groups

	L (left)	Numbers of mice	R (right)	Numbers of mice
OD	L occipital dorsal	2	R occipital dorsal	2
D	L dorsal	2	R dorsal	2
FD	L frontal dorsal	3	R frontal dorsal	2
F	L frontal	2	R frontal	3
FV	L frontal ventral	2	R frontal ventral	3
V	L ventral	4	R ventral	2
OV	L occipital ventral	2	R occipital ventral	2
O	L occipital	2	R occipital	2

10.1523/ENEURO.0094-23.2023.f1-1 Extended Data Figure 1-1 Images of 16 regional groups. In this study, the data were divided into 16 groups according to the group classifications shown in Table 1. Two slices are included for each group. Starting from the next page, the results of the 16 groups are summarized in panels ***a*** and ***b***, respectively. ***a***, Angle of the slices cut out, and the MRI cross-section is indicated by a black line. If the line appears to be a single line, it is the case that the angles of the slices coincide incidentally. In other panels ***b***, the left and center images, respectively, show the distribution of neurons on the electrode (left) and the position of the slice on the atlas (center). The different colors of the markers in the left figure refer to the different cortical layers, and the Allen Institute Atlas is depicted in the picture of the MRI cross-section on the right. The color of the marker distinguishes between subcortical or intracortical layers, but layer differences are not used in this study. In panels ***b***∼, the right panels represent figures depicting the correlations between the average firing rates during the 1/4 to 2/4 period and the 3/4 to 4/4 period of the recorded time series. Download Figure 1-1, TIF file.

The thickness of the cortical slices was 300 μm, and we used a multielectrode array (MEA) system (Maxwell Biosystem, MaxOne) for simultaneous measurement of electrical activity. During the measurements, we refluxed an artificial CSF (ACSF) solution saturated with 95% O_2_/5% CO_2_.

Also, we performed spike sorting (Spyking Circus software) on the time series obtained from the electrical measurements to identify ∼1000 neurons; the short distance spacing between the electrodes of the MEA system (15 μm) allows us to estimate the spatial position of the neurons very accurately. The parameters used in the spike sorting were the same as those in Kajiwara et al. (2021).

Before and after the electrical measurements, we measured 3D scans of the brain surface (Ide et al., 2019), and by comparing and superimposing the scan data with the magnetic resonance (MR) images recorded before the brain was extracted, we were able to accurately define the slice positions of individual brain regions with less than one slice resolution (Fig. 1*a*).

### Connectivity estimation and E/I categorization

In this study, after classifying neurons into inhibitory and excitatory, we estimated the presence or absence of connections and calculated the strength of connections for each neuron type. It is important to note that the classification of neurons must be done before determining connectivity, since the shuffled null data and the real data for excitatory and inhibitory synaptic connections must be compared separately. Transfer entropy (TE) is used to estimate connectivity (connected or not) and sorted local transfer entropy (SLTE) is used to classify neurons into inhibitory/excitatory (E/I), a further modification on transfer entropy. The method used in this study is basically based on the method previously (Kajiwara et al., 2021). The source code is also available on GitHub (https://github.com/ShimonoMLab).

**Figure 2.**
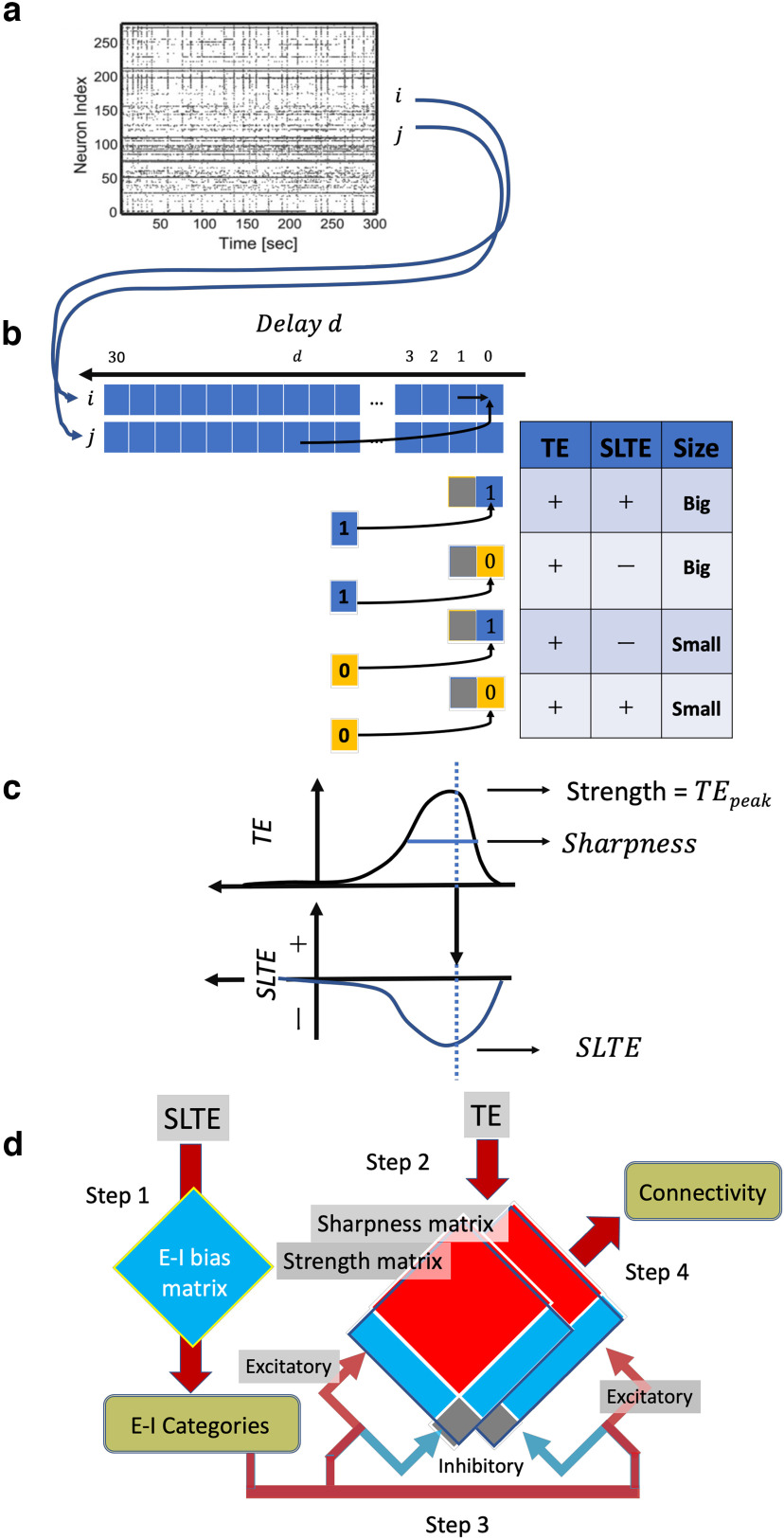
Estimation of E/I categories and intercellular connections from spike data. ***a***, Example of spike data displayed as a raster plot. Neurons i and j were extracted from this large number of neurons. ***b***, Then, the influence of other neuron j on the main neuron i was evaluated by transfer entropy. The effects of the combination of the activity state of neuron i at delay 0 and the activity state of neuron j at delay d on the TE and SLTE values were summarized in a table with positive and negative values. The magnitude of the effect was expressed as “big” or “small.” ***c***, TE was obtained as a function TE(d) that depends on delay, and from TE(d), the peak value strength and the peak sharpness were calculated. The value of SLTE at the delay where TE takes the peak was also extracted. ***d***, Flowchart describing the overall flow of cell categorization and connectivity estimation after the SLTE and TE are prepared. Step 1: the E-I bias matrix was obtained from the peak SLTE values, from which the E-I category for each neuron was determined. Step 2: strength and Sharpness were obtained as a matrix from the TE values for all combinations of neurons. Step 3: the matrix was classified into four connection types based on the E-I categories. Step4: finally, the presence or absence of connections and the strength of the connections were obtained by comparing them to the shuffled data for each of those categories.

TE used for connectivity estimation is a type of method to evaluate information transfer between different variables beyond false correlations and confounding factors (Okatan et al., 2005; Fig. 2*b*). Relationship between structure and function (Honey et al., 2007; Hlaváčková et al., 2009; Ito et al., 2011; Stetter et al., 2012; Timme et al., 2016), topology consistent with patch-clamp experiments, the presence of hubs and modules (Shimono and Beggs, 2014, 2015), are successfully demonstrated that distributions of connection weights are close to logarithmic (Nigam et al., 2016).

In other words, TE has its advantages both with respect to theoretical formulations and the requirements imposed by physiological data (Lizier et al., 2008; Vicente et al., 2011; Wibral et al., 2013).

In addition, SLTE, used to classify neurons into inhibitory and excitatory, is a measure like transfer entropy (TE), but sorts local transfer entropy according to the positive and negative signs of the different interactions (Lizier et al., 2008). Because it sorts by positive and negative sign, SLTE can distinguish excitatory and inhibitory interactions of presynaptic spike events relative to postsynaptic spike events using the opposite sign of the local transfer entropy of excitatory and inhibitory interactions (Lizier et al., 2008; Goetze and Lai, 2019).

### More information about connectivity estimation

The transfer entropy (TE) used for connection estimation measures the extent to which the prediction accuracy of the activity of neuron i is improved by including information on the spiking activity of neuron j, compared with prediction accuracy based solely on the past of neuron i (Fig. 2*a*). The equation for TE used in this study is expressed as follows:

(1) $${\text{TE}}_{\text{J}\to \text{I}}\left(\text{d}\right)=\underset{{\text{i}}_{\text{t}},{\text{ i}}_{\text{t}-1},{\text{ j}}_{\text{t}-\text{d}}}{\sum }\text{p}({\text{i}}_{\text{t}},{\text{ i}}_{\text{t}-1},{\text{ j }}_{\text{t}-\text{d}})\;\log\left(\frac{\text{p}\left({\text{i}}_{\text{t}}{\text{|i}}_{\text{t}-1},{\text{ j}}_{\text{t}-\text{d}}\right)}{\text{p}\left({\text{i}}_{\text{t}}{\text{|i}}_{\text{t}-1}\right)}\right).$$

This equation quantifies the expected value of the local transfer entropy $\log\left(\frac{\text{p}\left({\text{i}}_{\text{t}}{\text{|i}}_{\text{t}-1},{\text{ j}}_{\text{t}-\text{d}}\right)}{\text{p}\left({\text{i}}_{\text{t}}{\text{|i}}_{\text{t}-1}\right)}\right)$ for all ${\text{i}}_{\text{t}-1}$, ${\text{j}}_{\text{t}-\text{d}}$ and ${\text{i}}_{\text{t}}$ (Schreiber, 2000). The variables i or j are given the index of time to express if neuron i or j is active or not at a time bin. The values are 1 when they are spiking, and 0 when they are not spiking.

The size of the time bin was set to 1 ms, which is common for spiking data, and we evaluated the degree of improvement in prediction accuracy when the t−d ms time bin of neuron j was used in addition to the t−1 ms time bin of neuron i to predict the state of the most recent t ms time bin of neuron i (Fig. 2*b*). This is because it was demonstrated that, for neuron j, the inclusion of multiple past time bins does not significantly alter prediction accuracy as a function of TE(d) (Shimono and Beggs, 2015; Nigam et al., 2016; Fig. 2*c*).

When there is direct interaction because of synaptic connections from neuron j to neuron i, TE(d) shows a strong and sharp peak (Berry and Pentreath, 1976). Here, Strength is defined as the peak value of TE(d), so $\text{TE}({\text{d}}_{\text{peak}})$, and Sharpness is defined by the following equation (Fig. 2*c*; Shimono and Beggs, 2015; Nigam et al., 2016; Kajiwara et al., 2021):

(2) $$\text{Sharpness}=\frac{\sum_{\text{d}=0}^{\text{d}={\text{d}}_{\text{peak}}+\text{τ}}\;\mathrm{TE}(\text{d})}{\sum_{\text{d}=0}^{\text{d}=30}\;\mathrm{TE}(\text{d})}.$$

Here, the range of the denominator of Sharpness in Equation 2 is set to 30 ms as the time window as the sufficient length of time window for electrical signals to transmit in the spatial size of the brain region (1 × 2 mm) we record (Mason et al., 1991; Swadlow, 1994; Shimono and Beggs, 2015), and the numerator is calculated using the standard deviation of time delays between the spike of a presynaptic neuron and the subsequent spike of a postsynaptic neuron across all neuron pairs, with a set τ value of 4 ms.

From this raw data, we created a shuffled spike array by swapping the spikes of presynaptic neurons into spike-free bins within ±10 ms (Fig. 2*d*; Hatsopoulos et al., 2003). The TE obtained from it is called ${\text{TE}}_{\text{j}\to \text{i}}^{\text{shuffle}}$, and the TE obtained from the original data are called ${\text{TE}}_{\text{j}\to \text{i}}^{\text{real}}$. Then, by comparing the raw connectivity matrix with the connectivity matrix generated from the 100 shuffled spike series, we selected the connections with strong and sharp peaks in TE(d) on the Strength-Sharpness space. Here, we shuffled uniformly across the entire 30-ms time window. In this study, we used the same threshold as in Kajiwara et al., 2021 to determine the connections, and the connection probability was expressed as a median and standard deviation, resulting in 2.8 ± 3.3%.

The connection strength was defined as ${\text{IT}}_{\text{j}\to \text{i}}={\text{TE}}_{\text{j}\to \text{i}}^{\text{real}}-{\text{TE}}_{\text{j}\to \text{i}}^{\text{shuffle}}$ for the shuffled data. This connection strength has been repeatedly evaluated by comparing it to EPSPs measured by patch clamp recordings (Shimono and Beggs, 2014, 2015). See previous studies for details (Shimono and Beggs, 2015; Nigam et al., 2016; Kajiwara et al., 2021).

### More information about cell categorization

The mathematical definition of the sorted local transfer entropy (SLTE) used to classify neurons into inhibitory/excitable (E/I) is as follows:

(3) $${\text{SLTE}}_{\text{J}\to \text{I}}\left(\text{d}\right)=\underset{{\text{i}}_{\text{t}},{\text{ i}}_{\text{t}-1},{\text{ j}}_{\text{t}-\text{d}}}{\sum }\text{p}({\text{i}}_{\text{t}},{\text{ i}}_{\text{t}-1},{\text{ j }}_{\text{t}-\text{d}}){\left(-1\right)}^{\left({\text{i}}_{\text{t}}-{\text{j}}_{\text{t}-\text{d}}\right)}\log\left(\frac{\text{p}\left({\text{i}}_{\text{t}}{\text{|i}}_{\text{t}-1},{\text{ j}}_{\text{t}-\text{d}}\right)}{\text{p}\left({\text{i}}_{\text{t}}{\text{|i}}_{\text{t}-1}\right)}\right).$$

In SLTE, the local transfer entropy is weighted differently by the multiplier ${\left(-1\right)}^{\left({\text{i}}_{\text{t}}-{\text{j}}_{\text{t}-\text{d}}\right)}$. Depending on whether the events of each neuron i and j are spiking or inactive, i_t_ and j_t-d_ will be 1 or 0. Therefore, this multiplier is +1 when i_t_ and j_t-d_ are both the same event and −1 when they are different events (Fig. 2*b*). As a result, in the case of an excitatory interaction, the local transfer entropy is added, and in the case of an inhibitory interaction, the local transfer entropy is subtracted. This reordering rule results in the sum of all terms being positive for excitatory interactions and negative for inhibitory interactions. Note that in this quantification, for example, the amount of information given by $\text{p}\left({\text{i}}_{\text{t}-1}=0,{\text{j}}_{\text{t}-\text{d}}=1\right)$ for a rare event ${\text{i}}_{\text{t}}=1$ is much larger than that given by $\text{p}\left({\text{i}}_{\text{t}-1}=1,{\text{j}}_{\text{t}-\text{d}}=1\right)$ for a frequent event ${\text{i}}_{\text{t}}=0$ (Fig. 2*b*).

To emphasize the time window where direct synaptic influence is evident, we selected SLTE values with a time delay d in which TE exhibits a peak value (Fig. 2*c*). In the subsequent sections, the peak SLTE value will simply be referred to as the E-I bias.

Then, after removing the bottom 10% of the absolute value of the E-I bias to ensure a high signal-to-noise ratio, the E-I bias value was sorted to calculate the remaining top 90% of E-I bias, which was derived from the output side for each transmitting neuron separately based on the order of the peak TE values, ${\text{TE}}_{\text{nor}}={\text{ TE}}_{\text{peak}}/(\text{FR}\cdot \text{log }\left(\text{FR}\right)+\left(1-\text{FR}\right)\cdot \text{log}\left(1-\text{FR}\right))$, normalized to account for the effect of firing rate.

In the two-dimensional space of this sum and the logarithm of the per-neuron firing rate, we identified relevant clusters using hierarchical clustering based on the Ward variance minimization algorithm and assigned excitatory labels to neurons belonging to clusters that were relatively common and on the positive side of the SLTE sum, which is naturally predicted from the Dale’s principle (Dale, 1934).

In Kajiwara et al. (2021), we verified that the classification performance of this method is also high for computational models and *in vivo* data; we confirmed that similar classification performance can be obtained with SLTE alone, but in the end, the firing rate is the second axis, considering that the combination of SLTE alone gives slightly better classification performance than SLTE alone.

As a final summary, we list how the TE-based connection estimation and SLTE-based neuron classification can be combined as the following four steps procedures (Fig. 2*d*).

Step 1: first, from the SLTE, neurons are classified into excitatory and inhibitory neurons through the E-I bias matrix. Step 2: next, from the TE, the strength matrix (a matrix of peak intensities relative to delay) and the sharpness matrix (a matrix of peak sharpness relative to delay) are computed. Step3: the two matrices were then decomposed into four categories: excitatory-excitatory, excitatory-inhibitory, inhibitory-excitatory, and inhibitory-inhibitory. Step 4: finally, connectivity and connection strength were determined using shuffled null data for each of the four connection types. In this process, we partition the two-dimensional space of Sharpness and connection strength into a grid. We then identify the regions where the sample size of the original data exceeds that of the shuffled data by a certain proportion as the connected areas. Please refer to Kajiwara et al. (2021) for more detailed information.

### MRI acquisition

This study measured 3D T-weighted (T2W) images of the whole brain in each mouse. For this purpose, a 7T, 210-mm horizontal bore, preclinical scanner (BioSpec 70/20 USR, Bruker BioSpin MRI GmbH) MR system equipped with a 440 mT/m, 100-μs ramp time gradient system was used for relaxation enhancement (RARE) sequence was used; for RF excitation and signal reception, an orthogonal volume resonator [35 mm of inner diameter (i.d.), T9988; Bruker BioSpin] was used.

We also used a protocol called TurboRARE-3D (Bruker BioSpin), and the specific acquisition parameters are as follows: repetition time (TR) 2000 ms; echo time (TE) 9 ms; effective TE 45 ms; RARE factor 16; acquisition matrix size 196 × 144 × 144; field of view (FOV) 19.6 × 14.4 × 14.4 mm; acquisition bandwidth 75 kHz, axial (coronal direction in scanner setting): bandwidth 2.6-ms Gaussian π/2 pulse for fat suppression and spoiler gradient with 1051-Hz bandwidth, two dummy scans, averaging number 3, acquisition time 2 h 42 m, excitation pulse 2.59 ms, re-convergence pulse 1.94 ms, pulse shape: π/2 pulse, bandwidth 1051 Hz, fat suppression averaging number was 3. Pulse shape was sinc3, bandwidth was 2400 Hz. The software for the measurements was ParaVision 5.1. The cortical surface images were extracted from the measured MRI images using FSL. For details, see Ide et al. (2020).

### Slice preparation and electrophysiological recording

In this study, we recorded neuronal spikes from neocortical slices using the multielectrode array (MEA) system. For this purpose, mice were first sufficiently anesthetized with 1−1.5% isoflurane, then transferred a cortical slice to a Petri dish (100 × 20 mm) containing ice-cold cutting solution with air flow containing 95% O_2_ and 5% CO_2_. The extracted brain was cut into two blocks and then sliced into two to five slices (300 μm thick) in a diagonal angle to the cortical surface using a vibratome (Neo Linear Slicer NLS-MT; DOSAKA EM CO., LTD).

We selected cutting speed, frequency, and swing width as 12.7 mm/min, 87–88 Hz, and 0.8–1.0 mm, respectively, and slowed the speed down if the brain needed to be cut carefully for a temporary period.

All slices analyzed in this study were taken orthogonal to the cortical surface in any region (Fig. 1*b*). The angle cutting slices was carefully chosen, and the coordinates of the sections, including anterior-posterior coordinates and hemispheres, were recorded in a format when the brain sections were cut, and the slices were also reconfirmed and accurately recorded by embedding them within the MRI space acquired during the MR measurements described above (Ide et al., 2019).

The slices were incubated for 1 h in a beaker filled with prewarmed ACSF (∼34°C), then we selected one slice per animal and moved with a thick plastic pipette onto the MEA array and positioned the slice with a soft brush to record properly from a specific brain region including the cortex. The MEA array is rectangular in shape (2.0 × 4.0 mm), with 26,000 electrodes uniformly distributed (Fig. 1*c*) and the distance between adjacent electrodes was 15 μm (Maxwell Biosystem, MaxOne; https://www.mxwbio.com/).

Before the main electrical recording, a so-called prescan of 30 (s) was performed by recordings from adjacent 1020 sensors combinationally covering the all sensors, and up to 1020 sensor sensors that responded more strongly than 0.03 mV and more frequently than 0.1 Hz were selected.

The main electrical recording of spontaneous neural activity from the selected sensors was then performed for ∼2.5 h. In our experimental setting, the firing rate did not decay in this 2.5 h. Indeed, *in vitro* slice measurements with such prolonged recording times are rare. This long recording is an important factor in achieving high performances of connectivity estimation (Extended Data Fig. 1-1, right side panels, comparing ranked firing rates between 1/4–2/4 and 3/4–4/4 of the time series).

During this prescan and main measurements, the slices were still perfused at 1 ml/min with ACSF that was saturated with 95% O_2_/5% CO_2_ while controlling the temperature of the perfusate around 34°C.

### Brain surface scan

We recorded the brain surface in three different 3D scans: the whole brain immediately after extraction, the brain block cut into two blocks, and the brain block remaining after slicing into slices. For each object, the brain itself is measured at least twice, flipping up and down as needed. In addition, between one measurement from the same object, 16 images are scanned by rotating the object. For its 3D scanning, a scanning system based on 3D structural optical technology (SCAN in a BOX; Open Technologies) was used (Besl and McKay, 1992). Before scanning, it is extremely important to lightly wipe the brain surface with a microfiber cloth to prevent diffuse reflections (Ide et al., 2020). The scanned images were processed using the 3D scanning and processing software IDEA (including SCAN in a BOX).

First, the automatic alignment option of that IDEA was used to correct small disagreements between its 16 images and integrated them. The merged image was obtained for the number of times it was recorded from the same object.

Next, the merged images were superimposed and merged using the manual alignment option. The optimization algorithm was an iterative closest point (ICP) algorithm without nonlinear deformation, which is often applied to rigid 3D objects (Besl and McKay, 1992). After scanning, all scan planes were verified to be nicely overlapped.

Third, a high-resolution meshed object was created from the merged objects using the mesh generation option, saved in stl-binary format, and superimposed on the mesh image of the brain surface obtained from the individual MRI images using IDEA.

By the normalization process of FSL, the cross-sections of the superimposed brain slices are grouped together in the same normalized brain space (Extended Data Fig. 1-1*a*).

### Immunohistochemistry staining

The cerebral cortex has a “Baumkuchen”-like pattern of six overlaid sheets in the depth direction. Physiologically, each of these sheets are called layers. The layers are named layers 1–6 in order from the surface of the brain toward its depth. The layer architecture could be visually observed by immunostaining using NeuN and GAD.

To acquire information on cortical layers, immunohistochemical staining was performed in the 3 d after MEA recordings were completed; overnight after MEA measurements, brain slices were fixed with 4% paraformaldehyde (PFA) in PBS at 4°C.

On the next day (day 1), the same slices were washed with PBS for 5 min three times in total, and then incubated with antigen activating solution (10 mm sodium citrate, pH 6.0) for 20 min at 95–98°C. Then, after cooling it to room temperature (20–25°C), the slices were washed with 1% Na bisulfite in 50 mm Tris-buffered saline and incubated with blocking solution for 2 h at room temperature. The blocking solution is a solution prepared by adding 4% normal goat serum (NGS) and 0.5% octylphenol ethoxylate (Triton X-100) to a 1% Na bisulfite-Tris solution (pH 7.5). After washing with 1% Na bisulfite in 50 mm Tris-buffered saline, the slices were incubated with the blocking solution for 2 h at room temperature. The slices were then soaked in a primary antibody solution consisting of 1:800 mouse anti-GAD67 and 1:500 rabbit anti-NeuN and incubated overnight at 4°C. The primary antibody solution was prepared by diluting the primary antibody with 1% NGS and 0.5% Triton X-100 in 1% Na bisulfite-Tris solution.

On the second day, the same slices were replaced in a secondary antibody solution. The secondary antibody solution is a solution prepared by diluting a secondary antibody consisting of 1:500 goat anti-mouse and 1:500 goat anti-rabbit with Tris-NaCl solution (8.5 g/l NaCl in 50 mm Tris-buffered saline, pH 7.5) and Tris-NaCl with 3% NGS and 0.3% Triton X-100. Then, the slices were washed a total of three times for 5 min in Tris-NaCl solution and incubated overnight at 4°C.

On the third day, after washing a total of four times for 5 min in Tris-NaCl solution, the slices were placed on glass slides, embedded with antifade reagent (SlowFade Gold; Invitrogen), surrounded with coverslips, and then examined from the slides with a fluorescence microscope (All-in-One Fluorescence Microscope BZ-X710; Keyence) to record cell body distribution at 10× and 20× magnifications.

### Extracting layer categories

We applied Image processing consisting of three steps to the given fluorescence images (Kajiwara et al., 2021). Please refer the previous report in detail. Here, we briefly explain the method to define layer categories.

First, after applying noise removal preprocessing to the NeuN image, the cell bodies were identified using the Watershed algorithm. Here, the boundaries between neurons (including overlapping neurons) were first detected by drawing an estimated contour line that gradually increases from the centers toward the cell surfaces. From those detected boundaries, the centers of the neurons can also be defined.

Second, the number of neurons identified was divided by the spatial size of 50 × 50 pixels to calculate the density distribution. Along the depth direction, which is perpendicular to both the surface and the bottom of the cortex, a histogram proportional to the cell density was obtained. This was accomplished by summing the number of neurons in each bin along the direction perpendicular to the cortical layer.

Third, the layers are defined based on the density distribution of neurons. The neuron density distribution generally showed three convex points corresponding to the centers of layers 3, 4, and 6, and two concave points between those convex points. For that distribution, all boundaries were defined in the following three steps. (1) Between the cortical surface and the first convex point, we defined the boundary between layers 1 and 2 as the 1/3 point between the cortical surface and the first convex point, and the boundary between layers 2 and 3 as the 2/3 point between the cortical surface and the second convex point. (2) Next, the boundary between layers 3 and 4 was defined at the concave point between the first and second convex points. (3) Finally, the boundary between the layers 4 and 5 was defined at the midpoint between the second convex point and the second concave point, and the boundary between the layers 5 and 6 was defined at the midpoint between the second and third concave points. In this study, layers 2–6 are discussed because layer 1 protruded from the measuring region of some samples in the electrical measurement.

### Overlaying between a staining image and a MEA map

Next, we assigned layer labels to neurons derived from the electrophysiology by overlaying the layer boundaries obtained from the above image analysis on top of the multielectrode arrays.

In this procedure, first, the cortical layer boundaries obtained from the stained images were superimposed on the unstained images (photographic images taken immediately after MEA recording; Fig. 1*e*). Then, typical points (10 points on the cortical surface, seven points on the cortical base, and other additional points) common to the stained and unstained images were selected. Based on the iterative nearest neighbor algorithm (Greenspan and Godin, 2001), an optimal mapping of those typical points resulted in a transformation matrix from the stained image to the unstained one, which was then multiplied to the coordinates of the stained image to overlap the two images.

Second, to superimpose the unstained image onto the MEA coordinates, the four corners of the unstained image were selected, and a transformation matrix was obtained that optimally overlapped the MEA coordinates and the four points of the unstained image. By multiplying the inverse of the transformation matrix by the MEA coordinates, the layer boundaries obtained from the stained image were superimposed on the MEA, and layer labels were assigned to individual neurons (Extended Data Fig. 1-1*b*, left panels). In the subsequent analysis in this study, only the cortical areas were extracted and included in the analysis. This has the effect of reducing unexpected noise in the analysis of spiking data from neurons from outside of the cortex.

### Network metrics

#### Degree

Degree is a metric defined for each node and is the number of edges connecting to each node. In a directed graph with oriented edges, there are incoming edges and outgoing edges, so two indices, in-degree and out-degree, are defined for each type vertex. The in-degree is the number of edges coming into the vertex, and the out-degree is the number of edges going out from the vertex. The distribution of frequencies of vertices with similar degrees is called degree distribution and is frequently used to characterize structure of networks.

#### Weight

Weight refers to the strength of the connection, and here it is calculated by normalizing the amount of information calculated from the real spike data by the amount of information obtained when the time sequence of the spikes is shuffled. In other words, weight assesses the rate at which firing between two neurons occurs causally within a few milliseconds.

Past studies have reported that this metric shows statistically similar trends to synaptic connections (Song et al., 2005; Nigam et al., 2016; Kajiwara et al., 2021). It should be noted that if one neuron outputs more neurons, the amount of information sent to the individual destination neurons is smaller.

#### k-Core

Next, we used the k-Core index as another representative measure of centrality along with degree (Seidman, 1983). k-Core is determined by removing a node with zero connections (isolated vertex) to a node with one connection, to a node with two connections, and so on from the original connection structure. In general, a k-Core is defined as a partial network that consists only of nodes that (1) remain after removing nodes of order less than k, and (2) have a number of connections greater than k. To define k-Core in consideration of both rules (1) and (2), it should be noted that a k-Core does not necessarily correspond to a network that consists that of only nodes with degrees greater than or equal to k in many cases.

#### Feedback vertex set

A set of nodes in a directed graph (or network) is called a feedback vertex set (FVS) if removal of the incoming edges to the set eliminates all directed cycles. It is known that for reasonably wide classes of linear and nonlinear systems, statically and periodically steady states of the whole network are determined by specifying states of the nodes in an FVS (Akutsu, et al., 1998; Mochizuki et al., 2013).

The minimum feedback vertex set (MFVS) is the FVS with the minimum number of nodes and can be regarded as a minimum set of driver nodes. However, MFVS may not be uniquely determined in many cases. In order to cope with this issue, the weighted minimum FVS (WMFVS) was introduced by making use of weights assigned to nodes, where WMFVS is the MFVS with the maximum total node weight (Li et al., 2021). Recall that the weight of a node is defined as the sum of edge weights (connection strength) incoming to the nodes. Therefore, it is expected that WMFVS is uniquely determined if various weights are assigned to the nodes. Although computation of both MFVS and WMFVS is theoretically difficult (NP-hard), these can be computed efficiently for moderate size networks by using integer linear programming (Li et al., 2021).

## Results

**Figure 3.**
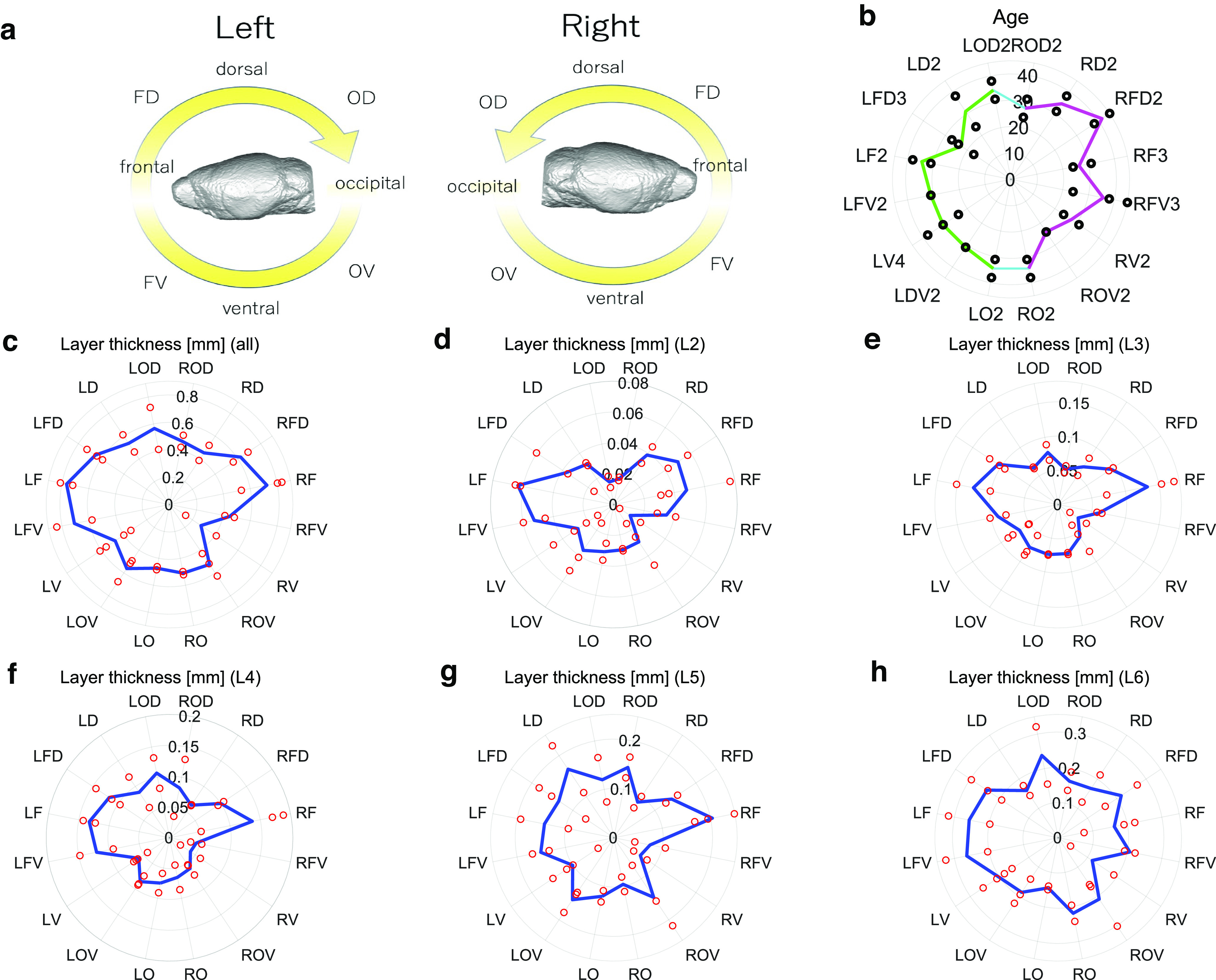
Radar charts of age and cortical thickness. ***a***, This panel expresses classification of the brain in 16 categories and their abbreviation names. Table 1 refers to these definitions of abbreviation names. In the following seven panels, the left hemisphere corresponds to the left half of the circle, and the right hemisphere to the right half. ***b***, This panel summarizes ages of mice used in individual experiments. Lines show the averages of the data within each of the 16 categories. (The left and right hemispheres are expressed with yellow-green and cyan colored lines, respectively.) Black points show the age of each individual mouse used in this experiment. ***c***, represents the thickness of all layers of the cortex, the line is the average value for each angle, and red dots express raw data. In addition to the panel ***c***, panels ***d–h*** express the thickness of individual layers (layers 2, 3, 4, 5, 6) separately. The pie chart regarding layer thickness is represented as a bar graph in Extended Data Figure 3-1. The data for cortical thickness are stored in Extended Data Table 3-1.

We aimed to compare the differences in functional networks between neurons in brain regions. Before that, we also compared basic physiological or anatomic metrics such as age, cortical thickness, and neuron densities. Therefore, we divided the brain into 16 circumferential categories based on the rotating angle as shown in Figure 3*a*, and defined abbreviated names as listed in Table 1. In this article, we simply call the 16 individual brain region categories as given names based on the angle.

10.1523/ENEURO.0094-23.2023.f3-1 Extended Data Figure 3-1 Bar graph representations of layer thickness. This figure is a redrawn representation of the pie charts of layer thickness, showcasing the observed significance in layer thickness, now depicted as bar graphs. The left column, consisting of panels ***a***, ***c***, ***e***, ***g***, ***i***, ***k***, represents the layer thickness redrawn as bar graphs. These are further categorized into two groups, “Frontal” and “Frontal Ventral,” versus all other regions, and are depicted as bar graphs in the right column, panels ***b***, ***d***, ***f***, ***h***, ***j***, ***l***. Download Figure 3-1, EPS file.

10.1523/ENEURO.0094-23.2023.tab3-1 Extended Data Table 3-1 Table summarizing the data of layer thickness presented in the Figure 3. Download Table 3-1, XLS file.

### Basic metric 1: age

Since the neuron density, firing rate, and network architecture are likely to change at different ages, it is necessary to measure all data for the same age group to simply compare between brain regions.

First of all, we show the age of individuals included in the 16 circumferential categories (Fig. 3*b*). The average age of each category shows that they are almost stable at around four weeks old (three to five weeks).

### Basic metric 2: layer thickness

Next, we observed some basic anatomic or physiological indices according to two steps. First, we compared the cortical thickness among the eight categories for individual hemispheres (Fig. 3*b*). Previous studies reported that cortical regions categorized as frontal group (or group F in our definition) are thicker than other regions (Grand'maison et al., 2013; Pagani et al., 2016).

Therefore, we performed the following statistical tests, hypothesizing that two to three adjacent brain regions around the frontal group would also show differences compared with other regions.

Here, we found a trend that the cortical thickness was higher in an adjacent pair of brain regions, categorized into the frontal and frontal dorsal groups, than other remaining seven groups (*p* < 0.005, Mann–Whitney test, groups F and FD). This trend was commonly observed between two hemispheres.

Second, we extended this finding further by observing the thickness of individual layers (Fig. 3*c–h*). Here, we found layer four was observed to be thinnest on the dorsal side, which corresponds to the motor cortex. This finding was also consistent with arguments from previous studies that stated layer four is not prominent in many regions, for example, the motor cortex (García‐Cabezas and Barbas, 2014; Yamawaki et al., 2014).

More interestingly, this trend of selectively increasing thickness in the F and FD groups is common across the many layers [layers 2–5, *p* < 0.05, Mann–Whitney test, effect size >0.5, sample sizes: (9,28)], except in the deepest layer [layer 6, *p* > 0.05, Mann–Whitney test, sample sizes: (9,28)]. Here, the statistical test was performed based on the same hypothesis for the case of all layers. We will discuss this new finding in the discussion section. Here, we performed a nonparametric test comparing the cortical thickness of the LF, LFV, RF, and RFV layers with that of the other 12 groups as the reference. Notice that we always used Mann–Whitney test corrected with Bonferroni’s corrections.

### Basic metric 3: density of active neurons

Next, we observed the neuron density. Density of active neurons is calculated by dividing the number of neurons with a firing rate >0.1 Hz by the recording area of the region where the neurons were present, but not divided by the thickness, 300 μm. We call the density of active neurons simply as “neuron density” in this report.

Here, notice that we produced acute slices that were orthogonal, and not tangential, to the brain surface. Therefore, the thickness of the cortex could correspond with one axis of our electrode array. Besides, we defined E/I (excitatory or inhibitory) categories for individual neurons based on properties of output connections of the neurons (Kajiwara et al., 2021). Therefore, we could calculate the density of active excitatory and inhibitory neurons by dividing the numbers of identified excitatory or inhibitory neurons by the spatial size covering a region of focus. Here, we put slices on a rectangular recording region to keep the cortical surface closely in parallel with one axis of the recording region. Therefore, for simplicity, we were also able to qualitatively use cortical thickness as the normalization factor instead of the size of the recording cortical region.

As mentioned before, we hypothesized that a few groups near the frontal region should be the main target of the statistical test to compare with other groups. This hypothesis was further supported by the observation that the frontal region exhibited a distinct cortical thickness.

Then, for the density of active excitatory neurons for all layers, frontal and frontal ventral regions showed significantly higher values than other regions [*p*-value = 0.0419 < 0.05, effect size = 0.63, sample sizes: (9,23), Mann–Whitney test].

Additionally, we performed a separate analysis of the individual layers defined based on immunostaining. The results showed that the trend of nonuniformity of density of active excitatory neurons was completely different between superficial and deep layers. Specifically, in deep layer 5 or 6, the high density around the frontal side was pronounced [layers 2–4, *p* > 0.05, sample sizes: (9,27)], while, in deeper layers, the high density around the frontal side was not so strongly observed [layer 5, *p* < 0.05, *p* = 0.035, effect size = 0.58, sample sizes: (9,27); layer 6, *p*-value = 0.015 < 0.05, F and FV, effect size = 0.67, sample sizes: (9,27), Mann–Whitney test]. Briefly speaking, the trend, that the density of active excitatory neurons is high around the frontal side was observed for all layers, but the trend was much stronger trend observed in the deep layers.

### Basic metric 4: E/I balance

We aimed to explore the differences between cortical layers in addition to obtaining a distinction between excitatory and inhibitory neurons.

**Figure 4.**
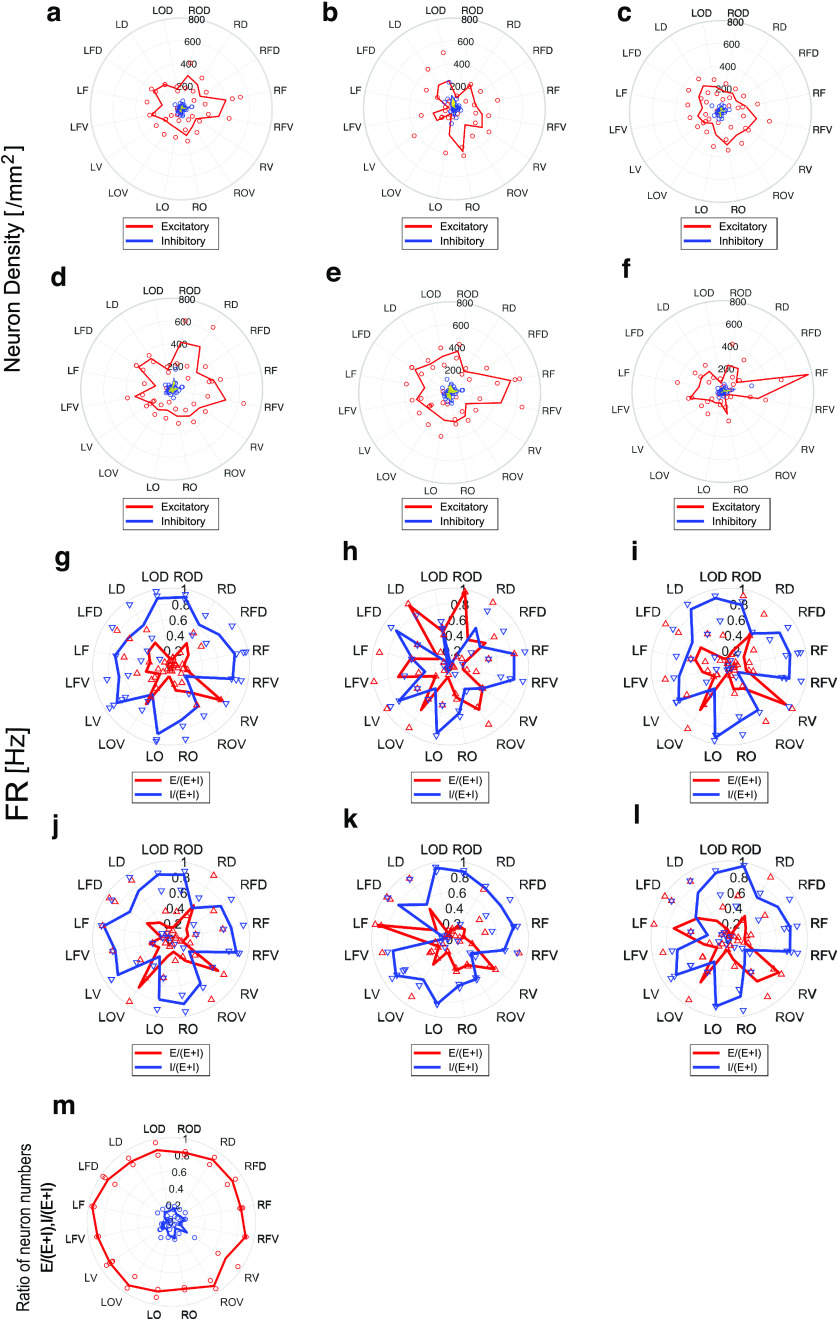
The most basic physiological metrics, neuron density, and firing rates. In all panels, features of excitatory and inhibitory neurons are shown as red and blue lines, and individual data samples correspond to dots. Panel ***a*** is the radar chart of averaged neuron density included in all layers of the cortical individual brain regions. Panels ***b–f*** separately express cell densities of individual layers (layers 2, 3, 4, 5, 6). Panel ***g*** summarizes averaged firing rate in all layers of the cortex, and the firing rates of individual layers (layers 2, 3, 4, 5, 6) are separately shown in panels ***h–l***. ***m***, E/I balance calculated as ratio of numbers of active excitatory or inhibitory neurons in the number of all active neurons at individual region groups. The pie chart regarding cell density is represented as a bar graph in Extended Data Figure 4-1. The data for neuron density and neuron count are stored in Extended Data Tables 4-1 and 4-2.

Past studies have shown that E/I balance is fairly stable and distributed in different areas of the cortex (Moreau et al., 2010; Xue et al., 2014). We quantified E/I balance as the ratio between numbers of active excitatory neurons and inhibitory neurons to the number of all active neurons. In fact, although the E/I ratio was stable (Fig. 4*m*), we observed that in the superficial layers, the neuron density of active neurons was higher in the occipital to dorsal occipital regions, whereas in the deeper layers, especially layer 6, the neuron density of active neurons tended to be higher in the frontal side. Notice that, in subsequent text, the “neuron density of active neurons” will be referred to simply as “active neurons.”

10.1523/ENEURO.0094-23.2023.f4-1 Extended Data Figure 4-1 Bar-graph representations of neuron density. This figure is a redrawn representation of the pie charts of neuron density, showcasing the observed significance in neuron density, now depicted as bar graphs. The left column, consisting of panels ***a***, ***c***, ***e***, ***g***, ***i***, ***k***, represents the neuron density redrawn as bar graphs. These are further categorized into two groups, “Frontal” and “Frontal Ventral,” versus all other regions, and are depicted as bar graphs in the right column, panels ***b***, ***d***, ***f***, ***h***, ***j***, ***l***. Download Figure 4-1, EPS file.

10.1523/ENEURO.0094-23.2023.tab4-1 Extended Data Table 4-1 Table summarizing the data of neuron density presented in the Figure 4. Download Table 4-1, XLS file.

10.1523/ENEURO.0094-23.2023.tab4-2 Extended Data Table 4-2 Table summarizing the data of neuron number used to plot the Figure 4. Download Table 4-2, XLS file.

Finally, we observed the firing rate in each brain region (Fig. 4*g–l*). This information beyond structural features is especially important because it is information that cannot be observed from dead slices by staining etc.

Surprisingly, although the firing rate of inhibitory neurons was basically higher than excitatory neurons, a bimodal peak in the firing rate of excitatory neurons was observed around the dorsal (LD, RD) and (occipital) ventral (LOV, RV) regions in both hemispheres. And after performing a statistical test between excitatory and inhibitory neurons, selecting the vicinity of the peaks as target, the firing rate of inhibitory neurons was not significantly greater than that of excitatory neurons [corrected *p* > 0.05, excitatory vs inhibitory neurons within dorsal and ventral groups, Mann–Whitney test, effect size: 0.74, sample sizes: (8,8)].

Up to this section, we have considered the properties of individual neurons. However, we also need to understand neurons as a network system, in which neurons essentially function by their mutual interactions. Therefore, from the next section, we start to analyze the topological properties as connected networks of nodes, neurons.

### Network metric 1: degree centrality

**Figure 5.**
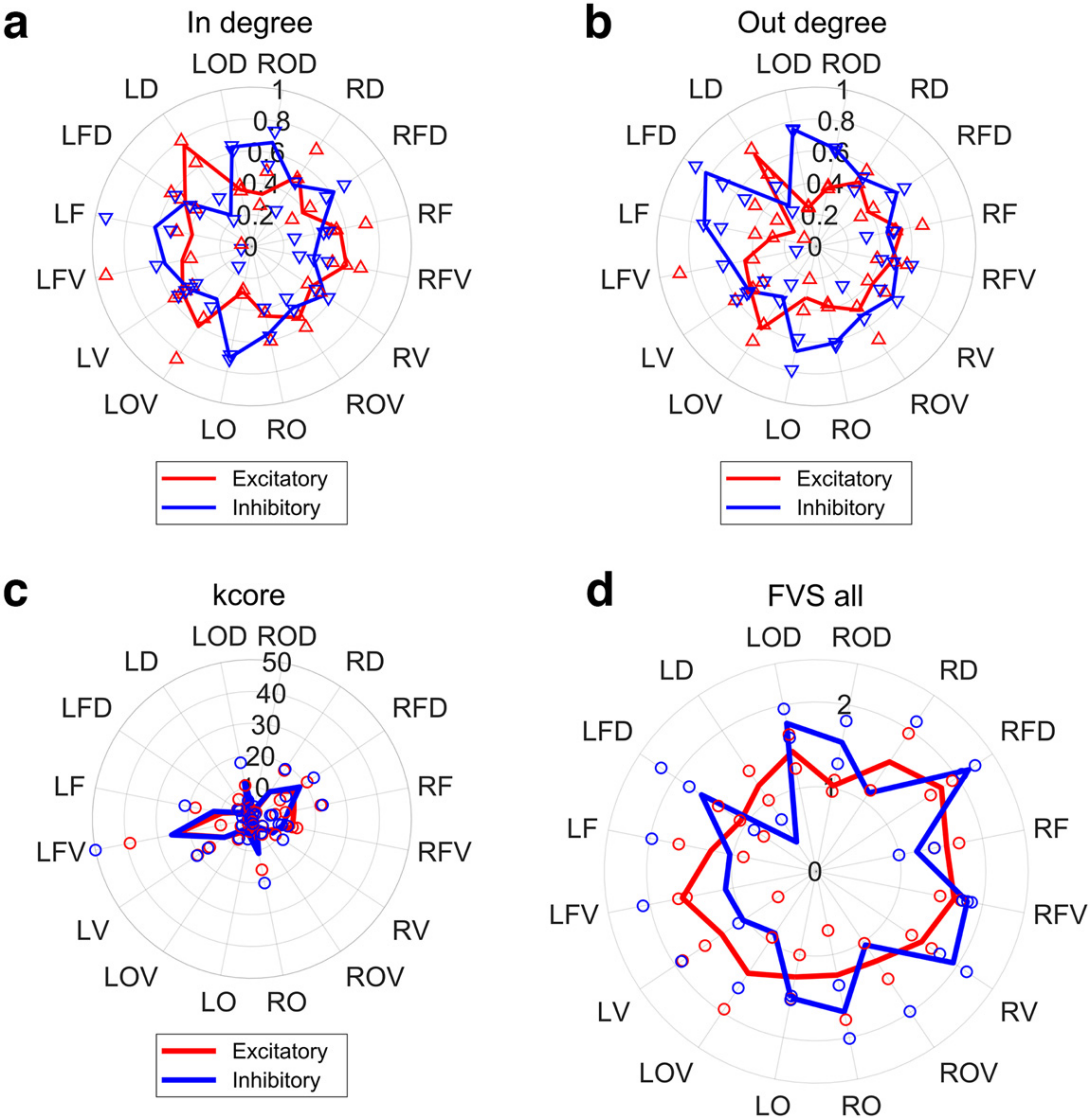
Basic topological properties at individual brain region groups. In panels ***a–d***, red and blue lines show features of excitatory and inhibitory neurons, respectively. ***a***, Number of inward connections, simply called in-degree. ***b***, Number of outward connections, called out-degree. ***c***, Same radar chart of k-Core centralities at individual brain regions. ***d***, Average FVS of all layers in the cortex. The meanings of colors of lines and dots are the same as Figures 2-4.

In order to move beyond the single neuron level, we will now explore several widely-used network measures (Fig. 5*a*,*b*). The degree centrality, a basic measure of how central a neuron is among possible paths within a network, was relatively higher in inhibitory neurons than in excitatory neurons in most of the regions, in terms both of in-degree and out-degree (in-degree: corrected *p*-value = 0.90, all regions, out-degree: *p* = 0.50, all regions).

It has been shown in previous studies that inhibitory neurons are functionally connected to more neurons than excitatory neurons in the somatosensory region (Kajiwara et al., 2021), and a similar trend could be observed at occipital regions (LOD, ROD, LO, RO). Current observation revealed that whether inhibitory or excitatory neurons show higher degree centrality largely depends on the cortical region observed.

### Network metric 2: k-Core centrality

In order to check whether the tendency observed with degree centrality can be shown with a different centrality metric, we also observed k-Core centrality (Fig. 5*c*). The radar chart of the k-Core of excitatory neuron groups showed that the value at the frontal side (RF, RFV, LF, LFV) is slightly larger than in other regions [corrected *p*-value = 0.16, Mann–Whitney test corrected by Bonferroni’s method, effect size: 0.60, sample sizes: (9,28)]. The following statistical tests are also corrected by the Bonferroni’s method.

### Network metric 3: controlling ability

Next, we used the feedback vertex set (FVS) index to measure the ability of inhibitory and excitatory cells to control other cells in each local circuit of the brain region. FVS provides a kind of measure of the ability of a neuron to change the intensity of its output while monitoring the surrounding situation by looping its own effects on other cells and returning to oneself.

Here, we will briefly explain how to calculate the FVS. In a directed network that can be written with arrows, we focus on a group of nodes. When removal of all incoming connections to the nodes in the group eliminates all directed cycles, the node group is called the feedback vertex set (FVS).

Furthermore, the FVS with the smallest number of nodes is called the minimum feedback vertex set (MFVS). Nodes in MFVS can be considered as driver nodes, and thus we can compare the control ability of the inhibitory neurons and the excitatory neurons by counting the number of MFVS nodes contained in each class of neurons. In our previous study, we evaluated the somatomotor region alone and found that inhibitory cells showed higher controlling ability than excitatory cells (Kajiwara et al., 2021). However, MFVS may not be determined uniquely. Therefore, in this study, we employed the weighted minimum FVS (WMFVS), which is the MFVS with the maximum total node weight, where the weight of a node is defined as the sum of edge weights (see below) incoming to the nodes.

In this study, somatomotor regions belong to LFD or RFD. As a result of this cortical wide-area evaluation, it became clear that the dominance of inhibitory to excitatory neurons’ controlling ability shows wide variety among regions (Fig. 5*d*). Here, we could observe relatively higher control ability of inhibitory neurons than excitatory neurons at LO, LOD, ROD, RO [corrected *p*-value = 0.40, Mann–Whitney test, corrected by Bonferroni’s method, effect size: 0.45, sample sizes: (8,8)].

### Network metric 4: weight

Weight is the strength of the connection. All observations up to this point were unweighted (not considering connection strength). And in those observations, the values for inhibitory neurons relatively more often exceeded those for excitatory neurons. We obtained the strength from the amount of information calculated from the spike data using a calculation procedure that shows properties similar to synaptic strength (Song et al., 2005; Nigam et al., 2016).

**Figure 6.**
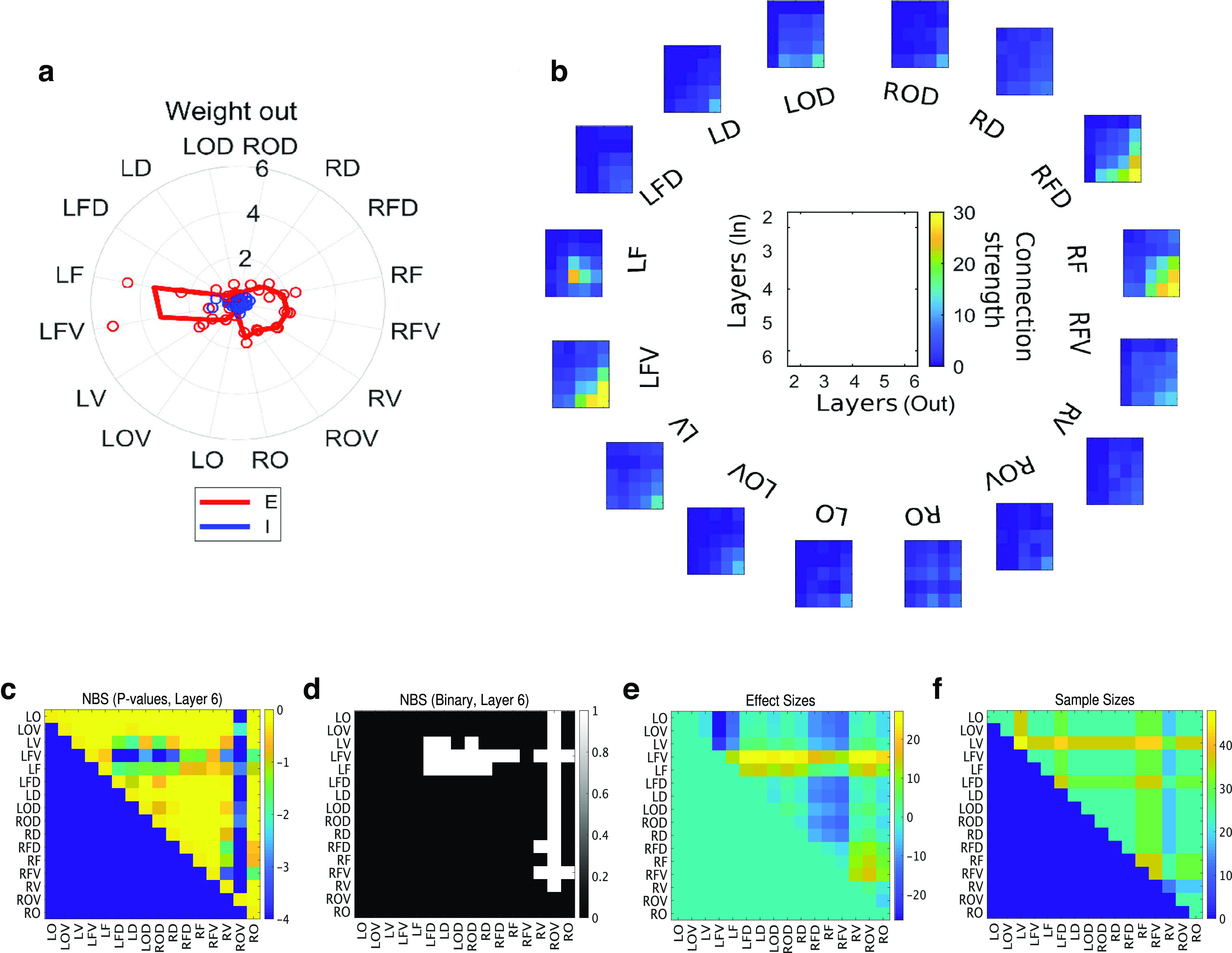
Interlayer connection strengths. ***a***, Radar chart of outward connection strengths. The strength of the inward connections also shows a similar pattern on the chart. The pie charts related to connection strength are also represented as bar graphs in Extended Data Figure 6-1. ***b***, Sixteen two-dimensional color maps evaluating the connection strength from input layers (horizontal axis) to output layers (vertical axis) are arranged in a circle. Sixteen maps correspond to the angles in Table 1 and many panels in Figures 2-5. When connections between input and output layers are strong (weak), they are colored yellow (blue). The blank figure placed at the center of the circle shows the meaning of the *x*- and *y*-axes commonly used in all color maps arranged in a circle, as well as the correspondence between color and intensity. ***c***, This is a color map of *p*-values obtained when testing the differences in connection strength between regions for all connections outputting from the sixth layer, based on Network-Based Statistics. ***d***, When applying a threshold of *p*-value 0.05, horizontal lines of low *p*-values centered around LFV and RFV can be observed. Vertical lines of low *p*-values from RV are also visible. ***e***, However, in terms of effect size, only the horizontal lines in frontal regions such as LFV, LF, RF, and RFV show high values. The results of the NBS test for the other layers are summarized in Extended Data Figure 6-2.

Next, we decided to observe connection strength (weight). When observing in terms of connection strength (weight), interestingly, the excitatory connections are reliably stronger than inhibitory connections across all cortical regions [corrected *p*-value = 2.5 × 10^−11^, Mann–Whitney test corrected by Bonferroni’s method, effect size = 0.76, sample sizes: (30,30)]. Moreover, the connection strength (weight) of excitatory neurons showed a pronounced strength at specific cortical regions, such as LFV, LF, RFV, and RF [Fig. 6*a*; corrected *p*-value = 0.0011, Mann–Whitney test corrected by Bonferroni’s method, effect size = 0.97, sample sizes: (7,7)].

10.1523/ENEURO.0094-23.2023.f6-1 Extended Data Figure 6-1 Bar-graph representations of connection weight. In panel ***a***, the pie chart regarding connection weights has been redrawn as bar graphs, and panel ***b*** is a representation of the same data but separated into two categories: “Frontal” and “Frontal Ventral,” versus all other regions, depicted as bar graphs. Download Figure 6-1, EPS file.

10.1523/ENEURO.0094-23.2023.f6-2 Extended Data Figure 6-2 Results of Network-Based Statistics for layers 2–5. This figure represents a set of three metrics: *p*-values, significant regions, and effect sizes, all evaluated for connection strengths based on Network-Based Statistics, as observed for connections from the sixth layer to other layers in Figure 6*c–e* in the main text. From top to bottom, they are arranged in order for layers 2–5. Download Figure 6-2, EPS file.

These regions are basically the frontal cortex. Recall that the activity quantified with firing rate was strongest in the motor cortex (or somatosensory cortex), which was slightly more dorsal (or occipital) than these areas.

Because current experimentally observed targets are segmented cortical slices, the projecting targets of individual neurons must exist within the observed region. This experimental setting means that the connection strength is defined as mainly and essentially the strength of connections within individual cortical regions. Therefore, simply speaking, we expect that the strong connection within a brain region leads to a strong firing rate at the same brain regions. However, the real resultsdemonstrated that the areas where strong connections were observed did not necessarily have a high firing rate.

Next, to observe the connections between different layers in more detail, we shifted to the observation as a weighted matrix where the horizontal and vertical axes are the indices of the layers.

### Network metric 5: interlayer weighted networks

We then observed the connection strengths (connection weights) observed in metric 4, grouped by layer. That is, the connection strength from each of layers 2–6 to each of layers 2–6 was represented as a single matrix for each region. In the individual color maps, the *x-axis* is for layers where the neurons sending out connections belong, and the *y*-axis is for layers where the neurons receiving connections belong. As a total, we were able to draw similar connection matrices from 16 regions for the left and right hemispheres (Fig. 6*b*), and the angles of 16 maps correspond to the angle used in other figures shown so far.

Furthermore, we conducted an evaluation of the connection strength for all combinations of regions concerning connections from the sixth layer (Fig. 6*c–e*). To provide a comprehensive assessment, we adopted the Network-Based Statistics (NBS) permutation test (Zalesky et al., 2010). In this test, the significance of the actual data are evaluated by comparing the *t* statistic of the real data with that of data obtained from random shuffling. This permutation process was repeated 10,000 times. Additionally, we calculated the effect size using the difference in means between the two samples.

As a result, we observed that *p*-values, particularly centered around frontal regions like LFV, LF, and RFV, and also in LOV, ROV, and RF, were significant in some cases (Fig. 6*c*,*d*). Upon examining the effect size, we found that among the significant regions, especially frontal areas like LFV, LF, RF, and RFV, showed relatively higher values (Fig. 6*e*). Although significant regions were visible in the vertical lines for ROV, the effect size was not observed to be particularly high.

From the above perspectives, we also attempted to test between the frontal and frontal ventral regions and other areas. As a result, in the sixth layer of the frontal and frontal ventral regions, we observed that both of output connections from sixth layers [corrected *p*-value = 2.4 × 10^−4^, Mann–Whitney test, effect size: 0.68, sample sizes: (60,156)] and input connections to sixth layers [corrected *p*-value = 0.040, Mann–Whitney test, effect size: 0.45, sample sizes: (24,72)] are stronger than the other layers in the frontal and frontal ventral regions which are located around the left and right edges of the figure (Fig. 6*b*, yellow regions).

Incidentally, in the NBS test, the tendency for significance to appear consolidated in the frontal region was observed in other layers as well. This suggests that in the frontal region, it does not appear to be a characteristic specific to the deeper layers. This point is further shown in Extended Data Figure 6-2.

## Discussion

In the Results section, we have observed differences among several cortical regions for each single metric. Now we consider the same results from a different perspective. Specifically, the results were reinterpreted by grouping the various metrics observed from the perspective of individual brain regions.

### Main findings

In this study, we conducted observations focusing on the frontal group. It has been reported in the past that cortical thickness is greater in this frontal region than in other regions (Fulcher et al., 2019). Furthermore, on observing the thickness of the cortex in separate layers, we found that the trend was observed in the superficial layer thicknesses but not in the deep layer ones.

It was also known in the past that the somatosensory cortex and frontal region are areas with high neuron density, following the visual cortex (Herculano-Houzel et al., 2013; Keller et al., 2018). We also separately observed neuron density by layers. As a result, the layer with significantly highest density in the frontal region was the deeper layer, while the visual cortex showed the highest density in the superficial layers.

In the counting of the number of neurons detected by staining, the neuron density was greatest in the visual cortex. However, in our results, the neuron density in the frontal cortex might exceed that in the visual cortex because we counted the sufficiently active neurons detected by our electrical activity measurement. In fact, the density of active neurons will not correspond with simple neuron density.

Interestingly, statistically, and most strongly, the connection strength was significantly higher around the frontal group although the number of functional connections between neurons was not significantly higher in the frontal group. Connection strength is regarded to be strongly related to response properties to external stimuli (Cossell et al., 2015). It is said that we can classify connection patterns, connecting to the frontal cortex, into multiple modes based on spatial patterns and connecting targets (Gao et al., 2022). Among the patterns, the wiring extending toward the medial side is especially regarded as a mode of connection pattern that supports the default mode network. In addition, the strong functional connections with the deep layer that we found would, in particular, function to strongly spread inputs, brought in via the spatial global wiring pattern, from/to the deep layers within the frontal area. In the near future, the simultaneous recording of local electrical flow in cellular resolution in the frontal region and of global electrical activity spreading along to the global wiring pattern will prove this suggestion more strongly.

The frontal region is also known to be a hub in the extensive network of cortical regions (van den Heuvel and Sporns, 2013; Liska et al., 2015), and also intensely interacts with subcortical regions (Oh et al., 2014). We can offer a possible interpretation of our findings in the context of the current literature. We suggest that the high density of highly active neurons and the strong connection strength in the depths of the network are maintained to serve as an edge (node) in the global network. In particular, we can expect the internal architecture plays an essential role in driving the default mode network observed in the rodent brain (Lu et al., 2012; Stafford et al., 2014; Coletta et al., 2020).

Recently, the relationship between the broad patterns of the default mode network and the cell-by-cell connections in each region have also been discussed (Whitesell et al., 2021). Our findings add scope to this relationship, given from dynamic spikes of living neurons, to elucidate the facts relating with these many other important findings.

### E/I balance

Next, interesting trends were observed in the regions labeled dorsal region and ventral region. These regions are more active with a relatively higher firing rate of excitatory cells than other regions.

These trends raise an intriguing hypothesis that these regions may play a role to generate high-firing neural activity in the mouse cortex. The slices labeled dorsal in this study are slightly closer to the occipital part of the top of dorsal side, and are located about halfway between the somatosensory and visual areas. It is known that when moving closer to the visual area from this position, the position becomes a slice that retains connections with subcortical thalamus (MacLean et al., 2006). It is important to note that in our results, the firing rate of excitatory neurons does not increase as one approaches the occipital visual area from the dorsal region. In other words, the higher firing seen in our dorsal area does not reflect the effect of the interaction loop with the thalamus, but rather the property of the internal local circuits.

From our results of E/I balance, we can say that the E/I balance is well maintained in cortical regions as a whole. There is a differential trade-off between speed and specificity in increasing the proportion of excitatory and inhibitory neurons (Sadeh et al., 2015). In this situation, optimal balance of excitatory and inhibitory synapses may allow an asynchronous irregular network to sustain a homeostatic state, which could be re-activated by external stimuli (Vogels et al., 2011). Such an arrangement could enable the system to generate sharp responses, to generate oscillations, and also to achieve optimal control gain and dynamic range (Isaacson and Scanziani, 2011). We used relatively young mice, three weeks old. This age range could reflect their importance in ongoing pattern formation in the process of developing neural circuits (Sadeh and Clopath, 2021). The experimental results in our study are consistent with the fact that, if we observe activities of living neurons as the whole of cortical regions, the contributions of excitatory and inhibitory neurons are highly balanced. At the same time, we need to notice that the E/I balance differs from region to region. How this imbalance helps effective information processes in individual brain regions will be an important future question.

### Limitations and future works

As described above, the present study has derived from the data some very interesting trends related to brain nonuniformity, which compensate for information that is not available from information on regional connections and structural cellular distribution previously provided from different recording modalities. However, since the scope of the analysis was limited within the cortex, it will be important in the future to develop the analysis to include subcortical regions as well. In fact, it is known that there are subcortical areas where cell densities are higher than in the cortex (Keller et al., 2018). More analysis of the same cortical data after parcellating with a cutting-edge atlas will also be important in the future to compare with results at more specific brain regions. It is also interesting to contrast the microconnectome obtained by our method with the structural microconnectome, which is obtained using SEM, etc. (Motta et al., 2019).

In fact, providing benchmark data with both structural and activity measurements while keeping the sample’s shape unchanged, such as in slices, is crucial for evaluating the accuracy of connection estimation. Sharing this information with readers is essential. It is also important to compare the results with the nonuniformity of genes expressed in the various brain regions (Mouse Genome Sequencing Consortium, 2002; Lein et al., 2007; Fulcher et al., 2019). This study took three years to accumulate more than two data points for individual 16 regional categories from three- to five-week-old mice, allowing us to check for repeatability in each area. Although significance was observed in the trends described above, it is important to increase the number of samples and to compare with data from older mice and compare between three-, four-, and five-week-old mice in the future beyond evaluating common properties within three- to five-week-old mice. However, it should be noted that the statistical significance of the results concerning the connection strength is exceptionally strong, and it is anticipated that the likelihood of significant changes even with an increase in sample size is low.

### Final remarks

In any case, there has been no study that systematically measures activity at ms temporal resolution from the whole cortex and discusses its activity and interaction network as in this study. Therefore, we expect the data and findings obtained in this study should be extremely valuable. This study may also be useful for improving future mathematical models of the brain and for advancing the systematic understanding of disease through animal models.

## Synthesis

Reviewing Editor: Arvind Kumar, KTH Royal Institute of Technology Decisions are customarily a result of the Reviewing Editor and the peer reviewers coming together and discussing their recommendations until a consensus is reached. When revisions are invited, a fact-based synthesis statement explaining their decision and outlining what is needed to prepare a revision will be listed below. The following reviewer(s) agreed to reveal their identity: Sacha van Albada. Note: If this manuscript was transferred from JNeurosci and a decision was made to accept the manuscript without peer review, a brief statement to this effect will instead be what is listed below.

Significance statement

Authors ask a very interesting and important question i.e. how the networks in different cortical regions differ? To address this question they have analysed neural activity, functional connectivity, layer thickness, neuron density, E/I ratio etc. from slices taken from 16 different cortical regions. This is valuable data. The main result that frontal regions are unique however requires further scrutiny. The reviewers have in general appreciated the work but they have also raised substantial concerns such as:

- Effect size and sample sizes (and sometimes statistical tests) are not reported. Usually it seems that the number is rather small and it is possible that some results may be spurious. Now, of course we are not asking for more experiments but authors should discuss which results are more robust and which might be spurious due to small sample size.

- Electrophysiology data is not shown. It is important to show how stable the activity was in different regions. The radar plots also do not reveal the variance of activity over time as well as over different slices.

- The hypotheses underlying the statistical tests should be explained clearly. In this regard one of the reviewers is concerned with the focus on the frontal region - this is not properly explained. And if left unexplained it may suggest that the hypothesis about frontal regions was formed when results were already known.

We think that these concerns can be addressed therefore we would like to invite you to revise the manuscript and address reviewers comments.

Detailed comments from the reviewers are appended below.

Reviewer #1

This manuscript characterizes basic connectomic and dynamical properties of local microcircuits and their variation across mouse cortex based on acute brain slices. Effective network interactions at the single-neuron level are inferred from spiking activity, and the frontal areas are identified as strong potential drivers of cortical activity.

The authors have done quite impressive experiments that are well suited to estimating the functional connectivity of the microconnectome. They used acute slices and long recordings (2.5h), which are ideal for such measurements. They also did microscopy and immunohistochemistry on the same slices, measuring many variables from the same system, resulting in a very valuable data set. However, there are a large number of issues in the analysis of the results, in spite of the well-suited experimental procedures. Furthermore, the writing and literature embedding should be improved. The overall sloppiness of the manuscript reduces the faith in the correctness of even the analyses and findings that have no obvious issues.

One issue is that the raw electrophysiological data are not shown anywhere, not even in the supplement. Thus, there is for instance no visual (or statistical, for that matter) verification of the lack of firing rate decay over the 2.5h recordings. Similarly, nothing is shown about the spike sorting process: neither the spikes nor the waveforms are shown. The description of the spike sorting should also be expanded with the software version and parameters, especially as the results can be quite sensitive to small parameter changes. This alone raises concerns about further analysis, since improper sorting would lead to invalid single-neuron results.

Moreover, the numbers of neurons detected in each area and layer and the numbers of significant E and I connections identified are not reported. Knowing to what extent the inferred functional connections adhere to Dale’s principle would say something about the validity of the methods. More generally, the value of the findings would be much enhanced by proper validation of the method with ground truth data at the level of individual connections (using ground-truth experimental data such as paired recordings, using simulations, or both).

Assuming proper spike sorting and identification of functional connections, the authors do perform some statistical tests on their results, but they never report effect sizes or the sample sizes that went into the test. In some cases the concrete p-values are not reported, while in others the specific p-value is stated, but not which test was used. To the authors’ merit, they do correct for multiple testing when analyzing the connectivity, since several different metrics are tested for the same data.

Another serious issue is that the analysis is focused on the frontal areas without a well-founded argument for this selection. Since the argument given (the thickness of frontal cortex) is not very plausible and first appears in the results, one wonders whether the hypothesis of frontal importance was formulated post hoc, after looking at the activity data - which would invalidate the numerical results of corresponding hypothesis tests. Since it would anyway be more informative and relevant to a wider audience to have results for all the regions, I think a corresponding descriptive analysis would be more appropriate.

In conclusion, in the current form, the paper unfortunately does not do the seemingly valuable data much justice.

In the detailed comments, I give a non-exhaustive set of points for improvement.

Abstract:

l. 10: “Especially” doesn’t really fit here.

l. 13: “enrolled” is not the right word here.

The last sentence of the abstract is too far-fetched and overstates the reach of the findings.

Significance statement: 16 are not “dozens”

“implies” is too strongly formulated in the last sentence

Introduction:

l. 45: It is not clear what ‘scales’ have to do with it at this point.

l. 60: I think more references on canonical (micro)circuits should be given here, especially including the earliest proposals on this (starting with Douglas, Martin & Whitteridge, 1989).

l. 64: “Collaborative” is not the right word here. I guess you mean “corroborative”.

l. 70: On the relationship between cell density and inter-regional connections, you may also cite work by Hilgetag et al./Goulas et al.

l. 73: It is unclear what is meant by “categories” here.

l. 82: It is not true that no previous work has compared the microconnectome across brain regions. See for instance Beul & Hilgetag (2015) Front Neuroanat.

Materials and Methods:

l. 122: ‘used in previous studies that have already been published studies’ → ‘used in previous published studies’ or similar

l. 152: A verb is missing in the sentence starting on this line.

l. 155 and 156 repeat each other.

p. 6 It would be interesting to know how the estimated connectivity compares with that inferred by other methods, such as that of Kobayashi et al. (2019) Nat. Commun.

l. 192-194: Please clarify if the labels or the values of i, j are 1 when there is a spike (it must be the latter, but the text says the former).

l. 197: Using only one past time bin for neuron I seems very little, representing only a minimal control for the possibility that I’s activity can be predicted from its past activity rather than from neuron J’s activity.

l. 199: The repeatedly cited Shimono & Beggs (2014) does not appear in the bibliography. It is also not clear to me which paper is meant - maybe the authors’ 2015 paper in Cerebral Cortex?

l. 205: It is not clear to me that 30 ms delays are appropriate to use in the denominator when such intervals can contain multiple spikes.

l. 209: Please specify what you took the standard deviation of.

l. 210: Were the spike trains only shuffled around identified TE peaks or across their entire length?

l. 235: Please clarify what you mean by “a time delay d in which TE exhibits a peak value”. Is d the time delay corresponding to the time at which the peak occurs?

l. 237: The lower 10% in absolute value?

l. 238: The top 90% for each sending neuron separately, or the top 90% across all neuron pairs?

It would be interesting to know how often individual neurons are inferred to send both excitatory and inhibitory connections according to the method used. This would give an indication of how error-prone the method is.

l. 279: Please specify what “it” refers to.

l. 284 Please check the units of the swing width.

l. 286: The orientation of the slices relative to the cortical surface is not evident from Fig. 1.

l. 295 and elsewhere: supramental → supplemental

l. 369: It is not the contour line that gradually increases.

l. 371: It is not the number of neurons that is smoothed.

l. 372-373: It is unclear what is meant by “the curves...were extended to a straight line”. Presumably, for determining the cell densities, the lines along the tops and bottoms of the slices should be such that just as much of the curves lies above and below the lines.

Extracting layer categories: It would be useful to illustrate the layer definitions with a figure. Also, it would be good to show that the definitions used are well-founded based on the literature. As is, they appear a bit ad hoc. Finally, providing all the layer thicknesses in a table (perhaps in the supplement) would enhance the usability of these results by others.

l. 435: There is an incomplete sentence at the end here.

l. 446: At this point it is unclear how weights are assigned to nodes.

Results

Fig. 3 caption: In Panels c-h, the dots are red, not black.

l. 540: Please clarify what you mean by “commonly between two hemispheres”.

Fig. 4: The plots of the inhibitory neuron densities are too small to be properly read off. Also, please give the units of the firing rate.

l. 566: The description of the slices as “coronal” does not appear to match the previous description as “oblique”. Do you mean orthogonal? Please clarify.

l. 573: Please indicate how you ultimately computed the density of active neurons: dividing by the size of the recording region, by the thickness, or both?

l. 582 It is unclear why a p-value is given for Layers 2-4 here and not for Layers 5 and 6.

It would be good to give the numbers and densities of active neurons in each region and layer in a table. This would increase the value of the data set for potential future users.

l. 593: It is unclear what is meant by E/I balance being “well mixed”.

The term “neuron density” referring to the density of active neurons could easily be misread as referring to an anatomical neuron density.

l. 604: Fig. 4g shows a peak of excitatory activity in region LOV rather than LV as written here.

Fig. 5 caption contains a note to self: “explain here what FVS means”.

l. 629-630: It is misleading to simply talk about the connectivity of inhibitory neurons as found by Kajiwara et al. (2021) as if this is anatomical connectivity, as the connections in that paper were inferred from activity as well (apparently using the same data and methods as described here).

l. 656: “controllability” → “controlling ability” (these are not the same: the former refers to being controlled)

Fig. 6: The incoming and outgoing weights appear to be identical. Perhaps a mistake was made in plotting here.

Fig. 6 caption: “When strong connections...” → “When connections...”

Also, the formulation with “from...to” makes it seem as if the x- and y-axes are swapped w.r.t. to the main text.

It is not true that frontal regions have high neuron densities (Herculano-Houzel et al. 2013; Erö et al. 2018). The authors seem to confuse density with total numbers of neurons. Furthermore, it is important to distinguish between 2D and 3D densities, as in terms of volume density, the highest neuron densities rather occur in visual regions.

l. 826: It is unclear what is meant by “two data points” here.

Reviewer # 2

Matsuda et al present an ambitious and technically sophisticated study attempting to quantify various anatomical and electrophysiological properties across the breadth of the cortex in mice. The text is generally clearly written and the figures are nicely presented.

Major comments

- The main limitation with this study is that the number of biological replicates is very low throughout. Many of the quantities estimated come from as few as n=2 mice, and the between-sample variance is substantial compared to the effect sizes. For example in Figure 3c it is claimed that the frontal brain regions have thicker cortex than other regions. But by eye it appears as though the difference within a given brain region is comparable to the difference in means across brain regions. These low sample sizes raise the possibility that some of the paper’s findings are spurious. Perhaps the authors can comment on the likelihood of this - or at least indicate which of their findings they consider most robust. The authors do acknowledge this aspect as a limitation at the end of the Discussion section, but the argument along the lines of ‘the data are time consuming to collect’ does not justify over-confident claims.

- A related issue is that of multiple comparisons during statistical testing. This paper contains a lot of measurements, and naturally it would be ideal to compare quantities between brain regions. However to test all possible comparisons would result in a lot of tests and potentially lead to false positive results. This issue does not arise directly in this paper as the authors often restrict their statistical tests to a small number of comparisons a priori (although they also occasionally do Bonferroni corrections). However the hypotheses underlying their choice of statistical tests is often stated without any further justification. It would be more convincing if the authors could elaborate on the rationale underlying each hypothesis, for example by referring to previous empirical studies, or logical arguments.

Minor comments

- Methods. The explanation of transfer entropy and the methodology for detecting synaptic connections is clearly described and the strengths of the measure are given. However what are the limitations of TE and SLTE? What factors may give rise to false positives or false negatives in their classification of connections? E.g. recording length, code parameter choices e.g. timebin size. It would be helpful for the reader to be given some insight on these limitations too.

- line 210. What percentile threshold was applied to the shuffled spike train measures to determine the cutoff for classifying if a connection was present or not?

- line 255 I am not sure what this sentence means: “Finally, connectivity and connection strength were determined using shuffled null data for each of the four connection types”. I can see how shuffling could be used to determine a cutoff for claiming sufficient evidence for the presence of a synaptic connection, but not how it could be used to determine connection strength?

- line 435. Something funny here, two extra words at the end of this line: “...greater than or equal to k. many cases.”

- Figure 3b caption says “The left and right hemispheres are expressed with red and blue colored lines, respectively.”, but the lines are green, cyan and magenta.

- Figure 3c caption says “black dots express raw data.”, I am guessing this should say “red dots”.

- line 536 implies that the authors may have pooled data across adjacent brain regions before performing statistical tests. But this is not stated explicitly. Can the authors explain how exactly they performed the statistical tests in the section “Basic metric 2: Layer thickness”, for example for the p-value reported on line 539?

- line 575 “Here, we hypothesized again that a few groups near the frontal region should be the main target of the statistical test to compare with other groups. “ Can the authors elaborate what this hypothesis was based on?

- line 582. I am not sure why there is a “(deeper?)” here.

- line 584. I am not sure that I understand what this sentence means: “briefly speaking, the trend, that the density of active excitatory neurons is high around the frontal side observed for all layers, is influenced by the same but much stronger trend observed in the deep layers.”

- Figure 4g-l. The units of firing rate are not indicated in the plot or caption - I am guessing Hz?

- line 604. I am unclear what exactly is being tested here. Is the underlying data for this statistical test plotted anywhere?

- Figure 5 caption says “(explain here what FVS means)”. Does this require elaboration or removal?

- line 684-685 I am unclear what aspect of this results this refers to: “However, the real results seem to hold a slightly discrepant trend.” Can the authors rephrase for clarity.

- line 749 says “As a basic property, we observed a symmetric contrast between the left and right hemispheres, whichever metric we examined.” Apologies if I missed this, but was this finding demonstrated via any statistical tests in the manuscript?

- line 791 says “these regions may be the source of energy that generates high-firing neural activity in the mouse cortex”. I am guessing that the authors do not mean energy here in the sense of ATP, mitochondria etc. If so it may be worthwhile rewording this to avoid reader confusion.

## Author Response

Synthesis of Reviews:

Computational Neuroscience Model Code Accessibility Comments for Author (Required):

Data analysis study.

Synthesis Statement for Author (Required):

Synthesis

The two reviewers are more or less happy with the revision but one point remains: The reviewers are concerned about the focus on the frontal areas. It is not about the importance of the frontal regions, it is more about leaving out other brain areas. When you have more brain regions the statistical significance threshold will of course be reduced and one wonders if the results would be still significant. In our opinion there is too much emphasis on doing some kind of hypothesis testing. Reviewers think that a hypothesis-free all-to-all- comparison. See more detailed comments in the report by the second reviewer. Also please ensure that the link is working.

Thank you for succinctly summarizing the key comments from the reviewers. In this paper, the most significant result appeared in relation to the connection strength. Therefore, we represented the connection strength as a matrix and applied Network Based Statistics (NBS). As a result, regions centered around the frontal area were selectively significant, allowing us to consolidate without changing the direction of the message. Could you kindly check again?

Reviewer #1

The authors have responded satisfactorily to my queries. In my opinion the paper is now acceptable for publication, apart from I could not access their provided GitHub link with their analysis code: https://github.com/Motoki878. Please correct before publication.

I apologize for the oversight. We have been using a GitHub page created by a previous student, and I had forgotten that we also cited it when we had other items to upload. I inadvertently changed the name. The current name is https://github.com/ShimonoMLab. Please check it.

Reviewer #2

The new supplements showing the recording locations and the correlation between mean firing rates at the beginning and the end of the recordings are greatly appreciated. This supplements strongly support the statement that the activity did not decay after 2.5h of recording.

Thank you for your understanding. We sincerely appreciate the feedback you provided.

Throughout the paper the authors used a polar plot to report the results, while this appears elegant in principle, it renders the reported values very difficult to compare. This problem is made worse now that the error values were included to the plots, in some cases it is not clear which error value corresponds to which area. Please report the values as bar plots with error bars, they are much easier to read.

Thank you for your comments. Regarding the thickness of the layers, cell density, and connection strength where significance was observed, we have added bar graphs replacing the pie charts in supplemental materials 3 and 4. I hope these figures will facilitate the readers’ understanding of the results.

Statistics:

Thank you for your valuable comments, especially regarding statistical tests and the issue of multiple comparisons. We have made essential revisions for your questions. Could you kindly share your important time to check it again?

The authors have now pointed out which test was used in all cases (Mann-Whitney U-test), but still do not report p-values in many cases nor the exact significance thresholds.

We feel there might be a misunderstanding here. Although, when the test results are not significant, we only mentioned the αvalue without stating the p-value, when the results are significant, we specified the p-values. The ‘Layer thickness’ section is an exception: even though it’s significant, we didn’t specify the p-value. This was because when summarizing the results from 2-5 layers in the text, including all values would make it very difficult to read. We made this exception with readability in mind. I hope you understand.

The authors focus their study in the differences between frontal areas and the rest of cortex, this choice still appears arbitrary and not grounded in the existing literature in all cases. The focus on frontal areas could be used to report fewer statistical tests, thus increasing the significance threshold(s) when applying the Bonferroni correction. It is not implied here that the authors deliberately restricted the analysis to increase the significance threshold. However, the fact that this would be technically possible vastly reduces the credibility of the results. Thus, in the current state I cannot recommend the publication of this paper, the authors should change the focus of the analysis.

　 Could you check the revisions in the following section?

The authors should report the tests between all areas (a table or array-like figure), as is done in an exploratory analysis, and thus correct the significance thresholds accordingly (roughly N = 16x(16-1)/2 = 120 tests, so the significance threshold should be alpha = 0.05/120 = 0.00041667). If after this analysis the frontal regions still appear significantly different from the rest, the authors’ claims would be much stronger. If the frontal areas no longer present systematic significant differences, the paper would still be completely valid and very valuable as an exploratory study, showing which areas in particular are similar and which are different. Considering the large scope of the experiments, finding no significant differences would also be a striking finding.

　　Thank you for your comments. Thanks to your input, we realized that Network Based Statistics is extremely effective in this context. We’ve added the results to Figures 6-(c-e), so please take a look. Also, as seen in Figure 6-(b), it’s evident that the intensity in the frontal region is weaker in the superficial layers compared to the deeper layers. However, when we defined the layers and compared them across regions, we found that layers 2-5 were also significantly stronger (see supplemental material 2). I’ve paid attention to this point and checked the expressions in the manuscript. If there are any issues with the phrasing, we would appreciate it if you could point them out.

Thank you for your careful comments despite your busy schedule.

I leave to the discretion of the editor whether this issue can be solved with a re-review or requires a resubmission.

If the revisions made this time are not sufficient, could you please notify us again?

We deeply appreciate you taking the time to help improve our manuscript despite your busy schedule.

## References

[B1] Akutsu T, Kuhara S, Maruyama O, Miyano S (1998) A system for identifying genetic networks from gene expression patterns produced by gene disruptions and overexpressions. Genome Inform Ser Workshop Genome Inform 9:151–160. 11072331

[B2] Batiuk MY, Martirosyan A, Wahis J, de Vin F, Marneffe C, Kusserow C, Koeppen J, Viana JF, Oliveira JF, Voet T, Ponting CP, Belgard TG, Holt MG (2020) Identification of region-specific astrocyte subtypes at single cell resolution. Nat Commun 11:1220. 10.1038/s41467-019-14198-8 32139688 PMC7058027

[B205] Berry MS, Pentreath VW (1976) Criteria for distinguishing between monosynaptic and polysynaptic transmission. Brain research 105:1–20. 175886 10.1016/0006-8993(76)90919-7

[B3] Besl P, McKay N (1992) Method for registration of 3-D shapes. Sensor fusion IV: control paradigms and data structures, pp 586–607. Bellingham: International Society for Optics and Photonics.

[B206] Bicks LK, Koike H, Akbarian S, Morishita H (2015) Prefrontal cortex and social cognition in mouse and man. Front Psychol 6:1805. 26635701 10.3389/fpsyg.2015.01805 PMC4659895

[B4] Carlén M (2017) What constitutes the prefrontal cortex? Science 358:478–482. 10.1126/science.aan8868 29074767

[B5] Chung K, Wallace J, Kim SY, Kalyanasundaram S, Andalman AS, Davidson TJ, Mirzabekov JJ, Zalocusky KA, Mattis J, Denisin AK, Pak S, Bernstein H, Ramakrishnan C, Grosenick L, Gradinaru V, Deisseroth K (2013) Structural and molecular interrogation of intact biological systems. Nature 497:332–337. 10.1038/nature12107 23575631 PMC4092167

[B6] Coletta L, Pagani M, Whitesell JD, Harris JA, Bernhardt B, Gozzi A (2020) Network structure of the mouse brain connectome with voxel resolution. Sci Adv 6:eabb7187. 10.1126/sciadv.abb7187 33355124 PMC11206455

[B7] Collins CE, Airey DC, Young NA, Leitch DB, Kaas JH (2010) Neuron densities vary across and within cortical areas in primates. Proc Natl Acad Sci U S A 107:15927–15932. 10.1073/pnas.1010356107 20798050 PMC2936588

[B8] Cossell L, Iacaruso MF, Muir DR, Houlton R, Sader EN, Ko H, Hofer SB, Mrsic-Flogel TD (2015) Functional organization of excitatory synaptic strength in primary visual cortex. Nature 518:399–403. 10.1038/nature14182 25652823 PMC4843963

[B207] Dale H (1934) Chemical transmission of the effects of nerve impulses. British Medical Journal 1:835. 20778253 10.1136/bmj.1.3827.835 PMC2445804

[B9] De Biase LM, Bonci A (2019) Region-specific phenotypes of microglia: the role of local regulatory cues. Neuroscientist 25:314–333. 10.1177/1073858418800996 30280638

[B10] DeFelipe J, Alonso-Nanclares L, Arellano JI (2002) Microstructure of the neocortex: comparative aspects. J Neurocytol 31:299–316. 10.1023/a:1024130211265 12815249

[B11] Douglas RJ, Martin KA (2004) Neuronal circuits of the neocortex. Annu Rev Neurosci 27:419–451. 10.1146/annurev.neuro.27.070203.144152 15217339

[B12] Douglas RJ, Martin KA, Whitteridge D (1989) A canonical microcircuit for neocortex. Neural Comput 1:480–488. 10.1162/neco.1989.1.4.480

[B15] Fulcher BD, Murray JD, Zerbi V, Wang XJ (2019) Multimodal gradients across mouse cortex. Proc Natl Acad Sci U S A 116:4689–4695. 10.1073/pnas.1814144116 30782826 PMC6410879

[B16] Fuster JM (2015) The prefrontal cortex. San Diego: Academic Press.

[B17] Gao L, et al. (2022) Single-neuron projectome of mouse prefrontal cortex. Nat Neurosci 25:515–529. 10.1038/s41593-022-01041-5 35361973

[B18] García‐Cabezas MÁ, Barbas H (2014) Area 4 has layer IV in adult primates. Eur J Neurosci 39:1824–1834. 10.1111/ejn.12585 24735460 PMC4201116

[B20] Goetze F, Lai PY (2019) Reconstructing positive and negative couplings in Ising spin networks by sorted local transfer entropy. Physical Review E 100:012121. 10.1103/PhysRevE.100.012121 31499780

[B21] Grand'maison M, Zehntner SP, Ho MK, Hébert F, Wood A, Carbonell F, Zijdenbos AP, Hamel E, Bedell BJ (2013) Early cortical thickness changes predict β-amyloid deposition in a mouse model of Alzheimer’s disease. Neurobiol Dis 54:59–67. 10.1016/j.nbd.2013.02.005 23454197

[B209] Greenspan M, Godin G (2001) A nearest neighbor method for efficient ICP. In Proceedings Third International Conference on 3-D Digital Imaging and Modeling pp 161–168.

[B22] Hama H, Kurokawa H, Kawano H, Ando R, Shimogori T, Noda H, Fukami K, Sakaue-Sawano A, Miyawaki A (2011) Scale: a chemical approach for fluorescence imaging and reconstruction of transparent mouse brain. Nat Neurosci 14:1481–1488. 10.1038/nn.2928 21878933

[B23] Harris KD, Shepherd GM (2015) The neocortical circuit: themes and variations. Nat Neurosci 18:170–181. 10.1038/nn.3917 25622573 PMC4889215

[B24] Hatsopoulos N, Geman S, Amarasingham A, Bienenstock E (2003) At what time scale does the nervous system operate? Neurocomputing 52–54:25–29. 10.1016/S0925-2312(02)00773-7

[B25] Herculano-Houzel S, Watson CR, Paxinos G (2013) Distribution of neurons in functional areas of the mouse cerebral cortex reveals quantitatively different cortical zones. Front Neuroanat 7:35. 10.3389/fnana.2013.00035 24155697 PMC3800983

[B200] Hlaváčková SK, Paluš M, Vejmelka M, Bhattacharya J (2007) Causality detection based on information-theoretic approaches in time series analysis. Phys Rep 441:1–46.

[B201] Honey CJ, Kotter R, Breakspear M, Sporns O (2007) Network structure of cerebral cortex shapes functional connectivity on multiple time scales. Proc Natl Acad Sci 104:10240–10245. 17548818 10.1073/pnas.0701519104 PMC1891224

[B26] Ide S, Kajiwara M, Imai H, Shimono M (2019) 3D scanning technology bridging microcircuits and macroscale brain images in 3D novel embedding overlapping protocol. J Vis Exp (147). 10.3791/58911-v 31132038

[B27] Isaacson JS, Scanziani M (2011) How inhibition shapes cortical activity. Neuron 72:231–243. 10.1016/j.neuron.2011.09.027 22017986 PMC3236361

[B202] Ito S, Hansen ME, Heiland R, Lumsdaine A, Litke AM, Beggs JM (2011) Extending transfer entropy improves identification of effective connectivity in a spiking cortical network model. PloS One e27431. 10.1371/journal.pone.0027431 PMC3216957 22102894

[B28] Kaas JH (ed) (2020) Evolutionary neuroscience. San Diego: Academic Press.

[B29] Kajiwara M, Nomura R, Goetze F, Kawabata M, Isomura Y, Akutsu T, Shimono M (2021) Inhibitory neurons exhibit high controlling ability in the cortical microconnectome. PLoS Comput Biol 17:e1008846. 10.1371/journal.pcbi.1008846 33831009 PMC8031186

[B30] Keller D, Erö C, Markram H (2018) Cell densities in the mouse brain: a systematic review. Front Neuroanat 12:83. 10.3389/fnana.2018.00083 30405363 PMC6205984

[B33] Lein ES, et al. (2007) Genome-wide atlas of gene expression in the adult mouse brain. Nature 445:168–176. 10.1038/nature05453 17151600

[B34] Li R, Lin CY, Guo WF, Akutsu T (2021) Weighted minimum feedback vertex sets and implementation in human cancer genes detection. BMC Bioinformatics 22:143–17. 10.1186/s12859-021-04062-2 33752597 PMC7986389

[B35] Liska A, Galbusera A, Schwarz AJ, Gozzi A (2015) Functional connectivity hubs of the mouse brain. Neuroimage 115:281–291. 10.1016/j.neuroimage.2015.04.033 25913701

[B36] Lizier JT, Prokopenko M, Zomaya AY (2008) Local information transfer as a spatiotemporal filter for complex systems. Phys Rev E Stat Nonlin Soft Matter Phys 77:e026110. 10.1103/PhysRevE.77.026110 18352093

[B37] Lu H, Zou Q, Gu H, Raichle ME, Stein EA, Yang Y (2012) Rat brains also have a default mode network. Proc Natl Acad Sci U S A 109:3979–3984. 10.1073/pnas.1200506109 22355129 PMC3309754

[B40] MacLean JN, Fenstermaker V, Watson BO, Yuste R (2006) A visual thalamocortical slice. Nat Methods 3:129–134. 10.1038/nmeth849 16432523

[B41] Mason A, Nicoll A, Stratford K (1991) Synaptic transmission between individual pyramidal neurons of the rat visual cortex in vitro. J Neurosci 11:72–84. 1846012 10.1523/JNEUROSCI.11-01-00072.1991 PMC6575189

[B42] Miller KD (2016) Canonical computations of cerebral cortex. Curr Opin Neurobiol 37:75–84. 10.1016/j.conb.2016.01.008 26868041 PMC4944655

[B43] Miller EK, Cohen JD (2001) An integrative theory of prefrontal cortex function. Annu Rev Neurosci 24:167–202. 10.1146/annurev.neuro.24.1.167 11283309

[B44] Mochizuki A, Fiedler B, Kurosawa G, Saito D (2013) Dynamics and control at feedback vertex sets. II: a faithful monitor to determine the diversity of molecular activities in regulatory networks. J Theor Biol 335:130–146. 10.1016/j.jtbi.2013.06.009 23774067

[B45] Moreau WA, Amar M, Le Roux N, Morel N, Fossier P (2010) Serotoninergic fine-tuning of the excitation–inhibition balance in rat visual cortical networks. Cereb Cortex 20:456–467. 10.1093/cercor/bhp114 19520765

[B46] Morishima M, Kawaguchi Y (2006) Recurrent connection patterns of corticostriatal pyramidal cells in frontal cortex. J Neurosci 26:4394–4405. 10.1523/JNEUROSCI.0252-06.2006 16624959 PMC6674016

[B47] Motta A, Berning M, Boergens KM, Staffler B, Beining M, Loomba S, Hennig P, Wissler H, Helmstaedter M (2019) Dense connectomic reconstruction in layer 4 of the somatosensory cortex. Science 366:eaay3134. 10.1126/science.aay3134 31649140

[B48] Mouse Genome Sequencing Consortium (2002) Initial sequencing and comparative analysis of the mouse genome. Nature 420:520–562. 10.1038/nature01262 12466850

[B50] Nelson SB (2002) Cortical microcircuits: diverse or canonical? Neuron 36:19–27. 10.1016/s0896-6273(02)00944-3 12367502

[B51] Nigam S, Shimono M, Ito S, Yeh FC, Timme N, Myroshnychenko M, Lapish CC, Tosi Z, Hottowy P, Smith WC, Masmanidis SC, Litke AM, Sporns O, Beggs JM (2016) Rich-club organization in effective connectivity among cortical neurons. J Neurosci 36:670–684. 10.1523/JNEUROSCI.2177-15.2016 26791200 PMC4719009

[B210] Oh SW, et al. (2014) A mesoscale connectome of the mouse brain. Nature 508:207–214. 24695228 10.1038/nature13186 PMC5102064

[B52] Okatan M, Wilson MA, Brown EN (2005) Analyzing functional connectivity using a network likelihood model of ensemble neural spiking activity. Neural Comput 17:1927–1961. 10.1162/0899766054322973 15992486

[B55] Pagani M, Damiano M, Galbusera A, Tsaftaris SA, Gozzi A (2016) Semi-automated registration-based anatomical labelling, voxel based morphometry and cortical thickness mapping of the mouse brain. J Neurosci Methods 267:62–73. 10.1016/j.jneumeth.2016.04.007 27079699

[B56] Sadeh S, Clopath C (2021) Excitatory-inhibitory balance modulates the formation and dynamics of neuronal assemblies in cortical networks. Sci Adv 7:eabg8411. 10.1126/sciadv.abg8411 34731002 PMC8565910

[B57] Sadeh S, Clopath C, Rotter S (2015) Emergence of functional specificity in balanced networks with synaptic plasticity. PLoS Comput Biol 11:e1004307. 10.1371/journal.pcbi.1004307 26090844 PMC4474917

[B215] Schreiber T (2000) Measuring information transfer. Physical review letters 85:461. 10991308 10.1103/PhysRevLett.85.461

[B58] Schröter M, Paulsen O, Bullmore ET (2017) Micro-connectomics: probing the organization of neuronal networks at the cellular scale. Nat Rev Neurosci 18:131–146. 10.1038/nrn.2016.182 28148956

[B60] Seidman SB (1983) Network structure and minimum degree. Social networks 5:269–287.

[B61] Shimono M (2013) Non-uniformity of cell density and networks in the monkey brain. Sci Rep 3:2541–2549. 10.1038/srep02541 23985926 PMC3756338

[B62] Shimono M, Beggs JM (2014) Network community, clusters and hubs in cortical micro circuits. BMC Neurosci 15:F2. 10.1186/1471-2202-15-S1-F2

[B63] Shimono M, Beggs JM (2015) Functional clusters, hubs, and communities in the cortical microconnectome. Cereb Cortex 25:3743–3757. 10.1093/cercor/bhu252 25336598 PMC4585513

[B64] Song S, Sjöström PJ, Reigl M, Nelson S, Chklovskii DB (2005) Highly nonrandom features of synaptic connectivity in local cortical circuits. PLoS Biol 3:e68. 10.1371/journal.pbio.0030068 15737062 PMC1054880

[B65] Stafford JM, Jarrett BR, Miranda-Dominguez O, Mills BD, Cain N, Mihalas S, Lahvis GP, Lattal KM, Mitchell SH, David SV, Fryer JD, Nigg JT, Fair DA (2014) Large-scale topology and the default mode network in the mouse connectome. Proc Natl Acad Sci U S A 111:18745–18750. 10.1073/pnas.1404346111 25512496 PMC4284535

[B66] Stetter O, Battaglia D, Soriano J, Geisel T (2012) Model-free reconstruction of excitatory neuronal connectivity from calcium imaging signals. PLoS Comput Biol 8:e1002653. 22927808 10.1371/journal.pcbi.1002653 PMC3426566

[B67] Susaki EA, Tainaka K, Perrin D, Kishino F, Tawara T, Watanabe TM, Yokoyama C, Onoe H, Eguchi M, Yamaguchi S, Abe T, Kiyonari H, Shimizu Y, Miyawaki A, Yokota H, Ueda HR (2014) Whole-brain imaging with single-cell resolution using chemical cocktails and computational analysis. Cell 157:726–739. 10.1016/j.cell.2014.03.042 24746791

[B211] Swadlow HA (1994) Efferent neurons and suspected interneurons in motor cortex of the awake rabbit: axonal properties, sensory receptive fields, and subthreshold synaptic inputs. J Neurophysiol 71:437–453. 8176419 10.1152/jn.1994.71.2.437

[B212] Timme NM, Ito S, Myroshnychenko M, Nigam S, Shimono M, Yeh FC, Beggs JM (2016) High-degree neurons feed cortical computations. PLoS computational biology 12:e1004858. 27159884 10.1371/journal.pcbi.1004858 PMC4861348

[B68] Uylings HB, Groenewegen HJ, Kolb B (2003) Do rats have a prefrontal cortex? Behav Brain Res 146:3–17. 10.1016/j.bbr.2003.09.028 14643455

[B69] van den Heuvel MP, Sporns O (2013) Network hubs in the human brain. Trends Cogn Sci 17:683–696. 10.1016/j.tics.2013.09.012 24231140

[B213] Vicente R, Wibral M, Lindner M, Pipa G (2011) Transfer entropy—a model-free measure of effective connectivity for the neurosciences. J Comput Neurosci 30:45–67. 20706781 10.1007/s10827-010-0262-3 PMC3040354

[B70] Vogels TP, Sprekeler H, Zenke F, Clopath C, Gerstner W (2011) Inhibitory plasticity balances excitation and inhibition in sensory pathways and memory networks. Science 334:1569–1573. 10.1126/science.1211095 22075724

[B71] Wang XJ (2020) Macroscopic gradients of synaptic excitation and inhibition in the neocortex. Nat Rev Neurosci 21:169–178. 10.1038/s41583-020-0262-x 32029928 PMC7334830

[B72] Whitesell JD, et al. (2021) Regional, layer, and cell-type-specific connectivity of the mouse default mode network. Neuron 109:545–559.e8. 10.1016/j.neuron.2020.11.011 33290731 PMC8150331

[B73] Wibral M, Pampu N, Priesemann V, Siebenhühner F, Seiwert H, Lindner M, Lizier JT, Vicente R (2013) Measuring information-transfer delays. PLoS One 8:e55809. 10.1371/journal.pone.0055809 23468850 PMC3585400

[B74] Xue M, Atallah B, Scanziani M (2014) Equalizing excitation–inhibition ratios across visual cortical neurons. Nature 511:596–600. 10.1038/nature13321 25043046 PMC4117808

[B75] Yamawaki N, Borges K, Suter BA, Harris KD, Shepherd GM (2014) A genuine layer 4 in motor cortex with prototypical synaptic circuit connectivity. Elife 3:e05422. 10.7554/eLife.05422 25525751 PMC4290446

[B76] Zalesky A, Fornito A, Bullmore ET (2010) Network-based statistic: identifying differences in brain networks. Neuroimage 53:1197–1207. 10.1016/j.neuroimage.2010.06.041 20600983

