# Supplementary figures and images for: Whole-Brain Evaluation of Cortical Microconnectomes
Source: eNeuro. 2023 Oct 25;10(10):ENEURO.0094-23.2023. doi: 10.1523/ENEURO.0094-23.2023 (PMC10616907; doi:10.1523/ENEURO.0094-23.2023)

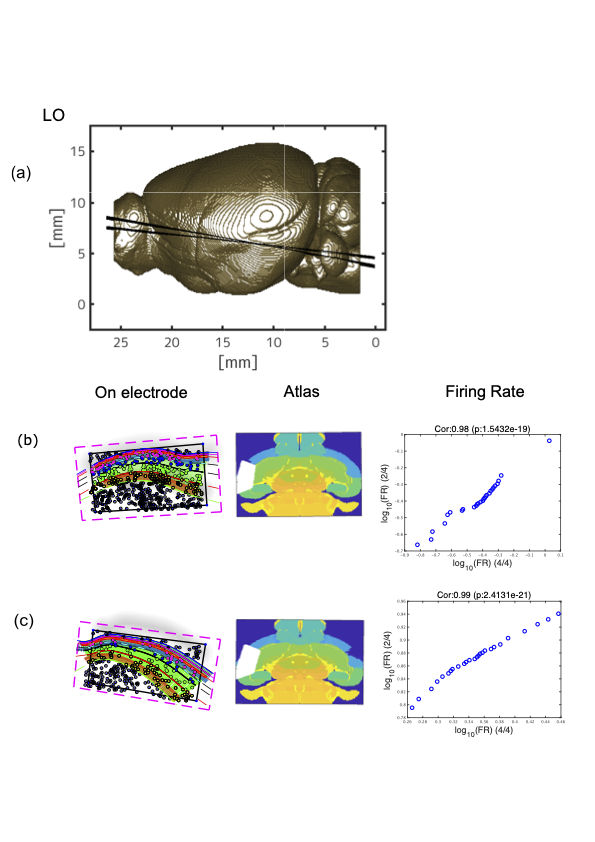

Supplement: Extended Data Figure 1-1 — Images of 16 regional groups. In this study, the data were divided into 16 groups according to the group classifications shown in Table 1. Two slices are included for each group. Starting from the next page, the results of the 16 groups are summarized in panels a and b, respectively. a, Angle of the slices cut out, and the MRI cross-section is indicated by a black line. If the line appears to be a single line, it is the case that the angles of the slices coincide incidentally. In other panels b, the left and center images, respectively, show the distribution of neurons on the electrode (left) and the position of the slice on the atlas (center). The different colors of the markers in the left figure refer to the different cortical layers, and the Allen Institute Atlas is depicted in the picture of the MRI cross-section on the right. The color of the marker distinguishes between subcortical or intracortical layers, but layer differences are not used in this study. In panels b∼, the right panels represent figures depicting the correlations between the average firing rates during the 1/4 to 2/4 period and the 3/4 to 4/4 period of the recorded time series. Download Figure 1-1, TIF file. [file enu-eN-TCFN-0094-23-s04.tif]
